# Genetic Architecture of Local Adaptation in Lunar and Diurnal Emergence Times of the Marine Midge *Clunio marinus* (Chironomidae, Diptera)

**DOI:** 10.1371/journal.pone.0032092

**Published:** 2012-02-22

**Authors:** Tobias S. Kaiser, David G. Heckel

**Affiliations:** Department of Entomology, Max Planck Institute for Chemical Ecology, Jena, Germany; American Museum of Natural History, United States of America

## Abstract

Circadian rhythms pre-adapt the physiology of most organisms to predictable daily changes in the environment. Some marine organisms also show endogenous circalunar rhythms. The genetic basis of the circalunar clock and its interaction with the circadian clock is unknown. Both clocks can be studied in the marine midge *Clunio marinus* (Chironomidae, Diptera), as different populations have different local adaptations in their lunar and diurnal rhythms of adult emergence, which can be analyzed by crossing experiments. We investigated the genetic basis of population variation in clock properties by constructing the first genetic linkage map for this species, and performing quantitative trait locus (QTL) analysis on variation in both lunar and diurnal timing. The genome has a genetic length of 167–193 centimorgans based on a linkage map using 344 markers, and a physical size of 95–140 megabases estimated by flow cytometry. Mapping the sex determining locus shows that females are the heterogametic sex, unlike most other Chironomidae. We identified two QTL each for lunar emergence time and diurnal emergence time. The distribution of QTL confirms a previously hypothesized genetic basis to a correlation of lunar and diurnal emergence times in natural populations. Mapping of clock genes and light receptors identified *ciliary opsin 2* (*cOps2*) as a candidate to be involved in both lunar and diurnal timing; *cryptochrome 1* (*cry1*) as a candidate gene for lunar timing; and two *timeless* (*tim2, tim3*) genes as candidate genes for diurnal timing. This QTL analysis of lunar rhythmicity, the first in any species, provides a unique entree into the molecular analysis of the lunar clock.

## Introduction

Biological clocks affect many physiological and developmental processes. They pre-adapt physiology to predictable changes in the environment and time important life history events to the most suitable occasions. Formally, biological clocks are described as consisting of (1) sensory *input pathways* that convey external information on time to synchronise (2) a central oscillating physiological process, the *oscillator*, which in turn controls (3) neuronal or hormonal *output pathways* that orchestrate rhythmic physiology and behaviour. The circadian clock, corresponding to the change of night and day, is probably the most wide-spread timing mechanism among organisms and certainly the best studied. It is the only biological clock for which the molecular basis is well understood [1], [2], [3]. Many marine organisms are not only affected by daily changes, but also by the tides, which recur about twice a day (every 12.4 hrs) and are modulated across the lunar cycle (29.53 days). Some species use the lunar cycle to synchronise reproduction within populations in the absence of distinct seasonality. Therefore, marine species often display tidal (12.4 hrs), lunidian (24.8 hrs), semi-lunar (14.77 days) or lunar (29.53 days) rhythms [4], [5], [6]. For a few of these rhythms it has been shown that they are not merely a direct response to cues in the environment, but have an endogenous basis, i.e. are controlled by a biological clock. The genetic and molecular basis of these tide or moon-related clocks is unknown, as are the possible interactions of these clocks with the circadian clock. A few recent studies have reported expression differences in circadian clock genes across the lunar cycle [7], [8], but it remains unclear if these expression differences are due to a lunar clock or due to differing nocturnal illumination.

The marine midge *Clunio marinus* (Chironomidae, Diptera) is a suitable model system to study both circadian and lunar clocks and their possible interactions [9]. *C. marinus* occurs in the rocky intertidal of the European Atlantic Coast from Spain to Norway. While the larvae need to be constantly submerged and settle at the lower fringe of the intertidal zone, the adults need the larval substrates to be dry for oviposition. This conflict has been solved by extremely reducing adult life-span to only a few hours and synchronizing adult emergence, mating and oviposition to the time when the water is as low as possible. These occasions predictably re-occur during the low tides of spring tide days, i.e. on the days just after full moon and new moon, during which the tidal amplitude is largest so that high tides are particularly high and low tides particularly low. Accordingly, larval development and pupation of *C. marinus* are characterised by a lunar rhythm that ensures that pharate pupae are only present around the spring tide days. Adult emergence is subject to a diurnal rhythm, making sure that on spring tide days the adults only emerge shortly before one of the two daily low tides. Both rhythms have been shown to be controlled by endogenous biological clocks [10], [11].

The tidal regimes differ for different places along the coast, and the diurnal and lunar rhythms of *C. marinus* populations along the coast are locally adapted [11], [12]. Previous studies have made use of these differences and assessed the genetic basis of the diurnal and the lunar rhythms in crossing experiments [12], [13]. The experiments involved laboratory strains from two particular populations of *C. marinus*, the *Jean* strain (from St. Jean-de-Luz, Basque Coast, France) and the *Por* strain (from Port-en-Bessin, Normandie, France). The strains differ in diurnal emergence time as well as in lunar emergence time. They also differ in the number of emergence peaks in one lunar cycle: while the *Jean* strain emerges only during the new moon spring tides (lunar rhythm), the *Por* strain emerges during both new moon and full moon spring tides (semi-lunar rhythm). This reflects different periods of the underlying circalunar clocks of these strains as measured in free-running experiments, i.e. experiments in which the strains are transferred from conditions with moonlight cues into constant conditions without moonlight cues. Under constant conditions without moonlight cues, the lunar rhythm in the *Por* strain continuous with a period of 13–14 days, the lunar rhythm of the *Jean* strain continues with a period of 26–27 days [11]. The timing differences between these two strains are stable and have been maintained in the laboratory for more than four years now (>12 generations), suggesting existence of a genetic basis to both diurnal and lunar timing.

An early crossing study reported genetic control of diurnal emergence time [13]. This was confirmed by our more recent experiments, and analysis of phenotype distributions in the old and the new backcross data indicated that the strain differences in the diurnal rhythm are most likely controlled by two independent loci [12]. We additionally found that lunar emergence time was under genetic control by two or more genetic factors. Moreover, the lunar and diurnal emergence times were not inherited independently, but showed a strong correlation in the backcross. As both the diurnal rhythm and the lunar rhythm are adapted to the local tidal regime of the source population, the change in the environmental factors imposing selection on both rhythms is highly correlated when moving along the coast. Thus, the genetic correlation of the phases of the two rhythms can be considered adaptive as well. One possible interpretation of this genetic correlation is that the same genes control both the lunar and diurnal rhythms. Alternatively, lunar and diurnal rhythms could be controlled by different genes, and selection might have shaped genome architecture towards genetic linkage in order to stabilise well adapted combinations of diurnal and lunar emergence times (see [12]).

The aim of this study was to identify the loci that control local adaptation in diurnal and lunar emergence times in *C. marinus* and to investigate the genetic basis of the correlation of the two traits. We constructed a genetic linkage map of the *C. marinus* genome using gene markers, microsatellites and amplified fragment length polymorphisms (AFLPs) and used the map to identify quantitative trait loci (QTLs) contributing to population differences in diurnal and lunar emergence times. In addition, we cloned and mapped several candidate genes (see Table 1) chosen for their known involvement in the circadian clock in other species, or their possible role in sensing moonlight as the *zeitgeber* (i.e. the time-setting agent) for the lunar clock. Our data not only shed more light on the genetic architecture of the intricate timing adaptations of *C. marinus*, but they also represent the first QTL map of lunar rhythmicity in any organism.

**Table 1:** Mapped candidate genes for the differences in diurnal (and lunar) emergence times between strains of *C. marinus* and their known functions in other organisms.

**Clock genes**	
period (per)	First clock gene to be identified [31]. Transcription factor in the core circadian clock, involved in a negative feedback loop in *D. melanogaster* [43].
cycle (cyc)	Transcription factor in the core circadian clock, involved in a negative feedback loop in *D. melanogaster* [44]. Homologous to *bmal* in mammals.
clock (clk)	Transcription factor in the core circadian clock, involved in a negative feedback loop in *D. melanogaster* [45], [46]. First discovered in mouse [47], [48].
timeless (tim)	Transcription factor in the core circadian clock, involved in a negative feedback loop in *D. melanogaster* [25], [26], [49]
timeless 2/timeout (tim2)	Involved in chromosome stability and light entrainment of the circadian clock in *D. melanogaster* [24].
timeless 3 (tim3)	Function unknown. Identified by sequence similarity to tim2. Similar genes are identified in *A. aegypti* and *C. quinquefasciatus* [this study].
cryptochrome 1 (cry1)	“Insect type” cryptochrome. Main photoreceptor of the circadian clock in *D. melanogaster* [50], [51].
cryptochrome 2 (cry2)	“Mammalian type” cryptochrome. Transcription factor in the core circadian clock of mammals [1]. Suggested to have similar function in non-drosophilid insects; absent from *D. melanogaster* [27], [52]
vrille (vri)	Transcription factor involved in a second feedback loop in the *D. melanogaster* circadian clock [53], [54], [55].
lark	Splicing factor that specifically affects timing of eclosion in *D. melanogaster* [56], [57]. Activates posttranscriptional *period* expression in mammals [58].
protein kinase mck1	Aka *glycogen synthase kinase 3* (*gsk3*) aka *shaggy* (*sgg*). Kinase known to affect the circadian clock period in *D. melanogaster* by phosphorylating the TIMELESS protein [59].
casein kinase 1 α (ck1a)	Regulates the circadian clock by phosphorylation of clock proteins [60], partially redundant to the casein kinases 1d, 1e and 2 [61], [62], [63].
**Photoreceptors**	
ciliary opsin 1 (cOps1)	Extraretinal photoreceptor in ciliary cells [22], [23]. Function unknown.
ciliary opsin 2 (cOps2)	Extraretinal photoreceptor in ciliary cells [22], [23]. Function unknown.
rhabdomeric opsin 2 (rOps2)	Photoreceptor, probably visual.

## Results

### A genetic linkage map for *Clunio marinus*

**Figure 1:**
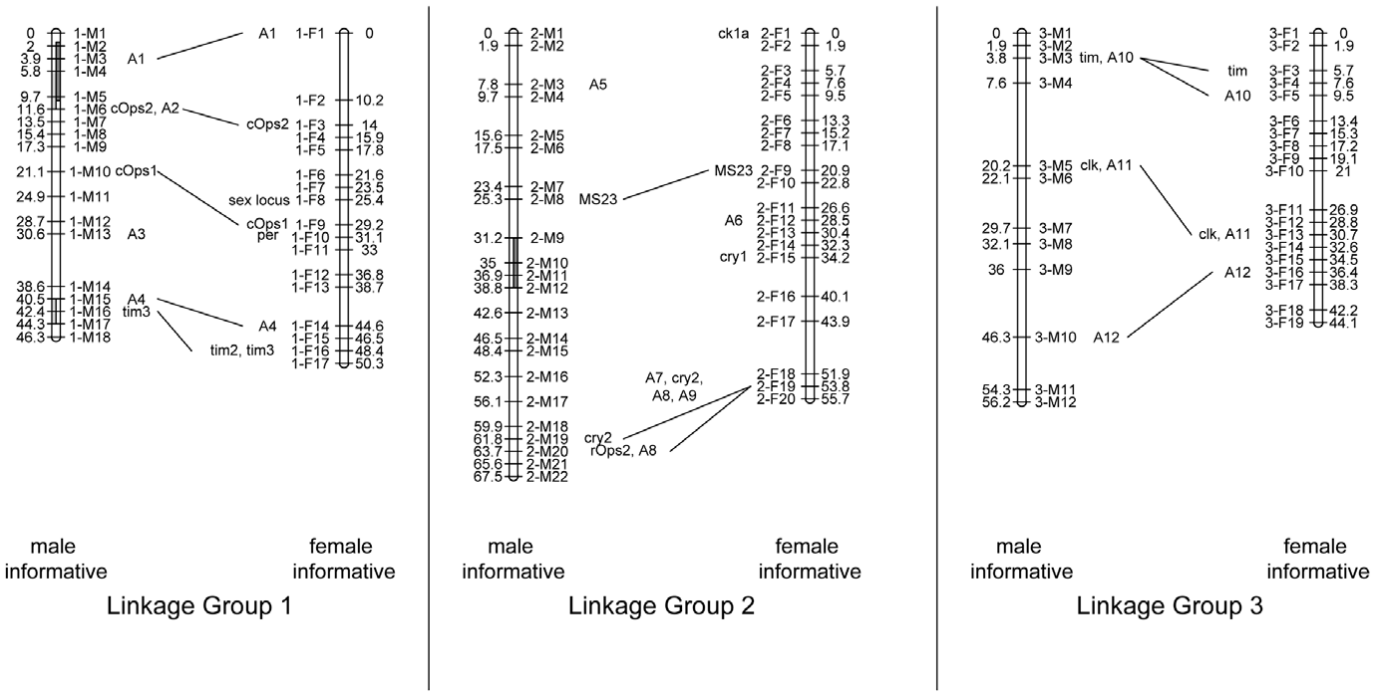
Male- and female-informative linkage map of the *Clunio marinus* genome. There are three linkage groups corresponding to the three chromosomes. Map length is in centimorgan (cM). Anchor loci (“A…”), light receptor and clock gene loci connect the two maps. The QTL for the differences in diurnal emergence time are shaded in light grey, the QTL for differences in lunar emergence time are shaded in dark grey.

The linkage map and the diurnal and lunar QTLs presented here are based on a previously reported [12] crossing experiment with the *Jean* and *Por* laboratory strains of the marine midge *Clunio marinus*. The largest backcross (BC) family (n = 54) of that crossing experiment was chosen to construct a linkage map based on gene markers, microsatellites and AFLPs (Table S1 and S2). Linkage map construction is based on the patterns of inheritance of the genetic markers in the BC progeny. Markers on the same chromosome are not inherited independently and therefore have similar patterns of inheritance, which allows the sorting of markers into linkage groups. Within linkage groups, marker patterns differ due to recombination events during meiosis, which in turn allows ordering the markers along the linkage groups. As the backcross progeny was equally informative for recombination in both the hybrid father as well as in the *Jean* strain mother, we constructed two independent maps for the genome (Fig. 1). A number of gene markers were mapped on both maps as anchor loci (Table S3), in order to be able to compare the two maps. After combining markers with identical patterns into marker groups, the two linkage maps contain 52 male informative marker groups spanning 170.0 cM and 56 female informative marker groups spanning 150.1 cM respectively. Genome length L was estimated to 192.1 cM for the male informative map and 167.2 cM for the female informative map. On the basis of the estimated genome length, 93.3% of the genome lies within 5 cM of a marker on the male informative map; for the female informative map the estimate is 96.5%. Thus, the genome is well-covered by the two linkage maps.

#### Estimated genome size

Flow cytometry was employed to obtain an estimate of the genome size of *C. marinus*. In this particular application of flow cytometry, tissues and cells are disrupted and nuclei are stained with a UV dye. The nuclei are then run through a capillary and pass a UV detector one by one. The UV signal is directly proportional to the DNA content of a nucleus and thus to the size of the genome. Genome size is presented as C value, which is the DNA content of a haploid nucleus given in picogram (pg). By comparing to organisms with known genome size, *C. marinus* nuclei were found to be about half the size of those of *Drosophila elegans* and about 0.95 fold the size of those of *Drosophila subobscura*. Comparison with the published C values of 0.2 pg for *D. elegans* [14] and 0.15 pg for *D. subobscura* [15] leads to two slightly inconsistent estimates for *C. marinus*, namely 0.1 pg and 0.14 pg. The transformation of C value into number of nucleotides depends on the AT content of the genome and possible base modifications, and a C value of 1 pg corresponds approximately to 976 Mb of DNA sequence [16]. Thus, we can roughly estimate that the *C. marinus* genome sequence is 95 to 140 Mb long. Following from the higher estimate of 140 Mb and the estimated genome lengths, one cM on the male-informative map corresponds on average to 0.73 Mb or less; and on the female-informative map, to 0.84 Mb or less.

#### Positions of candidate genes

**Figure 2:**
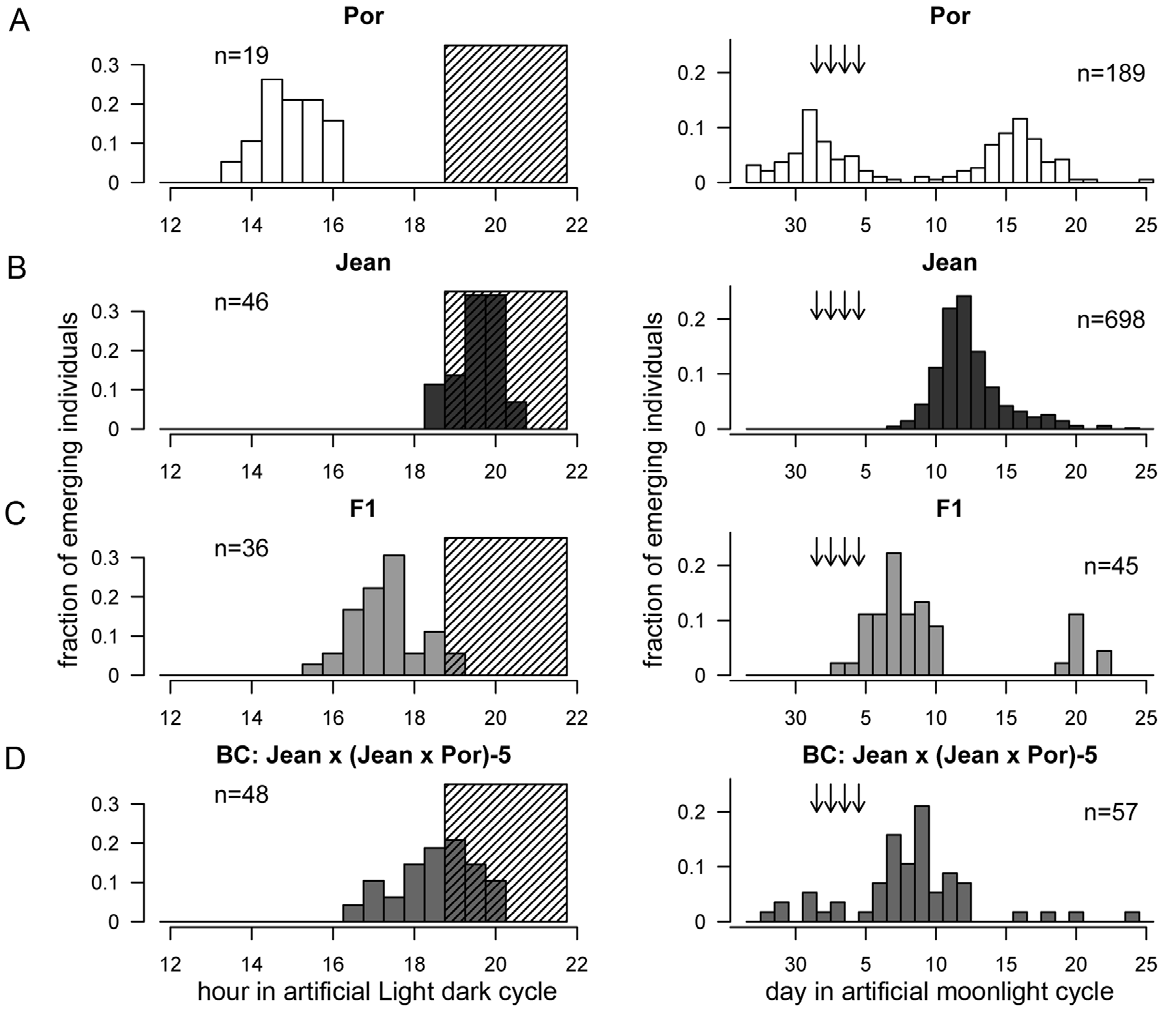
Lunar and diurnal emergence patterns of the parental strains (A, B), the F_1_ hybrids (C), and the BC family Jean×(Jean×Por)-5 (D) in the crossing experiment as reported previously [**12**] **.** The lunar rhythm is plotted as the fraction of individuals that emerged during each day of the artificial moonlight cycle. Arrows mark the days with artificial moonlight. Data of two lunar cycles are added up. The diurnal rhythm is plotted as the fraction of individuals emerging in 30 minute intervals across the artificial LD cycle. The shaded area indicates the dark phase. Individuals with imprecise diurnal emergence times (see methods) are not included in this graph.

As our ultimate interest is the identification of the genes underlying timing adaptations in *C. marinus*, *C. marinus* homologues of genes known to be involved in the circadian clock or light perception in other insects were cloned as candidate genes and mapped based on sequence polymorphisms (Table 1; GenBank Accessions JQ011261–JQ011276). Their positions are indicated along the linkage groups in Fig. 2 by the abbreviation of the gene name (as given in Table 1). For the genes *cycle, mck1* (alias *gsk3;* alias *shaggy* in *Drosophila melanogaster*), *vrille* and *lark* the F_1_ individual and the BC parent were identical heterozygotes, so that only about 50% of the BC individuals (namely the homozygotes) were informative for the mapping. The approximate positions of these genes on the male and female informative map were obtained by scoring the two classes of homozygotes: *cycle* is within the marker groups 3-M3 or 3-M4 on the male informative map and 3-F9 or 3-F10 on the female informative map; *mck1* belongs to marker group 2-M20 on the male informative map and to 2-F18 or 2-F19 on the female informative map; *vrille* is within 1-M10 to 1-M13 and 1-F10 to 1-F13; *lark* is within 3-M2 to 3-M3 and 3-F3 to 3-F4.

#### Synteny comparisons with other dipterans

To test if the *C. marinus* genome shows any conserved patterns of synteny with other dipterans (i.e. shared structure or gene order along the chromosomes), for all gene loci on the linkage map the *Drosophila melanogaster* and *Anopheles gambiae* homologues were retrieved from GenBank, and their chromosome arm locations were compared to the positions on the *C. marinus* linkage map (Table S4). There is no detectable pattern of conserved synteny overall. Only linkage group 3 in *C. marinus* seems to contain genes that are mainly found on chromosome 3 in *D. melanogaster* (5 of 6) and chromosome arm 2L in *A. gambiae* (3 of 4). The other linkage groups of *C. marinus* contain a random assembly of genes that are found on chromosomes X, 2 and 3 in *A. gambiae* or *D. melanogaster*. Only in one case – on linkage group 2 – a sequence of four consecutive genes in *C. marinus* is found on one chromosome arm in *A. gambiae*. However, the order of these four genes is not conserved between the two species.

#### Sex locus

*C. marinus* does not have distinct sex chromosomes [17], similar to other species in the family Chironomidae [18]. Instead, it must be assumed that there is a sex determining locus on one of the three autosomes. On the female informative linkage map of the *C. marinus* genome, marker group 1-F8 consists of four AFLP markers that are completely sex linked and thus must be close to the sex determining locus. The fact that they occur on the female informative map, and that no male-informative markers cosegregated with offspring sex, indicates that in *C. marinus* the females are heterogametic with respect to this sex-determining locus. There is only one previous report of female heterogamety in Chironomidae [19].

### The quantitative trait loci (QTLs) for diurnal and lunar emergence times

Quantitative trait locus (QTL) mapping compares that pattern of inheritance of a phenotype of interest in a mapping family to the patterns of inheritance of the genetic markers that were scored for the same mapping family. If there is a statistically significant similarity of the patterns of inheritance of the phenotype and a certain genetic marker, one assumes that a gene controlling the phenotype must be near that particular marker in the genome. By assessing many genetic markers along the genome, it is possible to scan the whole genome for loci that contribute to a phenotype, without having to make any a priori assumptions on candidate genes.

#### Trait values

The trait values for the parental strains, the F_1_ and the backcross (BC) were published previously [12]. For better understanding of the mapping approach they are briefly recapitulated here (Fig. 2, Table 2). For the BC only the family used for the mapping is given (Fig. 2D). The parental strains differ in diurnal emergence time by 4.6 hours (Table 2). Regarding the lunar emergence rhythm, the parental strains differ in both the phasing of the rhythm and the number of peaks in the artificial moonlight cycle (Fig. 2, A and B). The Jean strain has a lunar rhythm (one peak in an artificial moonlight cycle) while the Por strain has a semi-lunar rhythm (two peaks in an artificial moonlight cycle). Both the F_1_ and the BC family show two peaks in an artificial moonlight cycle, but the two peaks contain highly unequal numbers of individuals (Fig. 2, C and D). From the fact that the major peak of the F_1_ is intermediate in phasing between the single peak of the Jean strain and the first peak in the Por strain, we inferred that the first Por peak represents the physiological equivalent to the Jean peak. Based on this inference, the parental strains differ in the phasing of the lunar rhythm by 11.2 days (Table 2).

**Table 2:** Phenotypic data for the parental, the F_1_ and the BC generations.

	Phase in LD cycle (hours; mean ± SD (n))	Phase in artificial moonlight cycle, major peak (days; mean ± SD (n))
P: Jean	19.8±0.6 (46)	13.4±2.4 (698)
P: Por	15.2±0.7 (19)	2.2±2.3 (91)
F_1_	17.6±0.8 (36)	8.4±1.8 (34)
BC: all families	18.7±0.9 (67)	10.5±2.7 (68)^a^
BC: Jean×(Jean×Por)-5	18.8±1.0 (48)	10.2±2.9 (50)^a^

a These values differ slightly from those published in Kaiser et al. (2011) as one individual which was originally assigned to the minor peak based on its phenotype, was now identified to belong to the major peak based on its genotype (see methods).

#### QTL for the phase of the diurnal emergence rhythm

**Figure 3:**
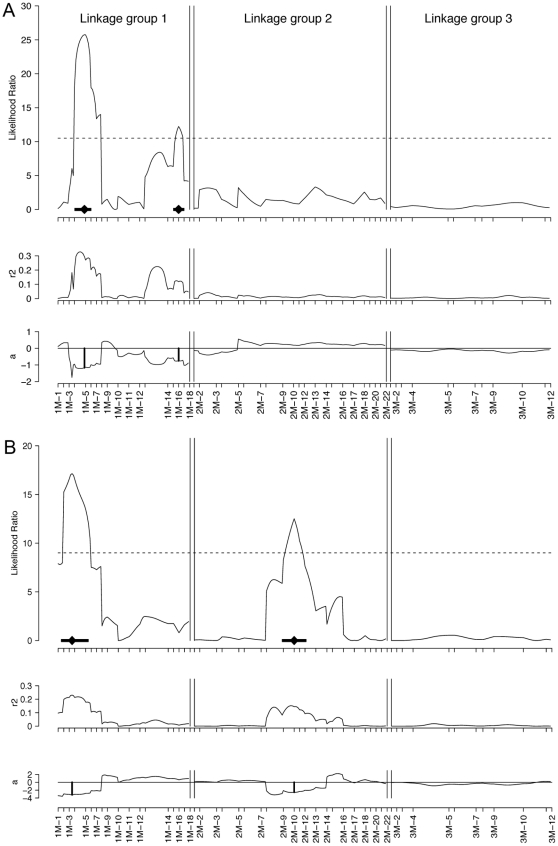
Composite interval map for differences in diurnal emergence time (A) and differences in lunar emergence time (B). The upper panel gives the likelihood ratio. The significance threshold (dashed line) was determined by bootstrapping (1000 replications). The diamond is the estimated QTL location, the black bar represents the one LOD score confidence interval. The middle panel gives the proportion of variance explained by the QTL (r^2^). The lower panel gives the estimated additive effects (a) in hours for diurnal emergence time and in days for lunar emergence time.

Composite Interval Mapping (CIM) based on the diurnal phenotypes of the backcross lead to the identification of two QTL detected at the two ends of the first linkage group (Fig. 3a), at locus 1-M5 and 1-M16, separated by 17 observed recombination events in 54 individuals (0.315). The two loci are not fully independent. Locus 1-M5 explains 29% of the variation in the diurnal phase of emergence; locus 1-M16 explains 12%. Their additive effects are 1.17 hours and 0.75 hours respectively, accounting for more than 85% of the difference between the F_1_ and the Jean strain.

#### QTL for the phase of the lunar emergence rhythm

Composite Interval Mapping (CIM) based on the lunar phenotypes of the backcross led to the identification of two QTL (Fig. 3b). One lies on linkage group 1 close to locus 1-M4, its confidence interval overlapping with the diurnal QTL at 1-M5. The other is on linkage group 2 at locus 2-M10. They explain 23% or 14% respectively of the variation in lunar phase of emergence. Their additive effects are 3.18 days and 2.53 days, adding up to 5.71 days and thus accounting for the full difference between the F_1_ and the Jean strain.

#### Potential locus controlling lunar vs. semilunar rhythm

The pattern of lunar emergence shows a minority of F_1_ and BC progeny emerging in a second peak, suggestive of a *Por*-like semilunar rather than *Jean*-like lunar rhythm for these individuals. All BC progeny in the minor peak were observed to inherit the same region on linkage group 2, ranging from marker group 2-M16 to 2-M22, from the *Por* grandparent. Unfortunately the genetic basis to this phenomenon is unclear, as the parental *Jean* strain sometimes also shows second peaks that are elicited by the highly artificial moonlight stimulus in the laboratory (for a detailed discussion see [12]). But if genetic control of the second peak were to be confirmed, the genetic factors controlling the trait would necessarily be in this region. As the number of peaks in a lunar cycle likely depends on the free-running period of the lunar clock ([11]; see introduction), this locus might control the period of the lunar clock.

#### Genetic correlation of the rhythms

**Figure 4:**
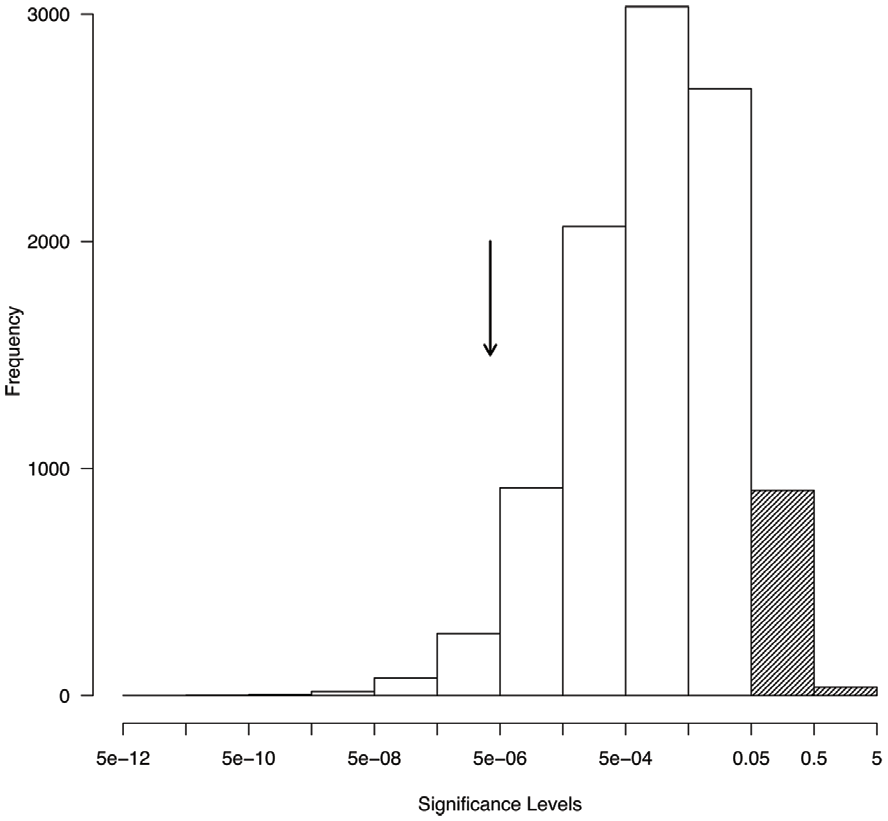
Distribution of p values of the expected correlation of lunar and diurnal emergence times as obtained in a re-sampling procedure based on the genetic architecture revealed by QTL mapping. The shaded bars give the fraction of non-significant p values. The arrow marks the p value of the observed BC distribution.

The original crossing experiment revealed non-independent inheritance of the diurnal and lunar phase phenotypes in the BC generation [12]. To test whether the distribution of QTLs as identified here can explain this correlation, a re-sampling procedure was applied. Briefly, starting from the observed phenotypes in the parental and F1 generations, assuming the genetic architecture as identified by QTL analysis and applying the rules of Mendelian genetics, the phenotype distributions of 10.000 expected BC generations were sampled. They were compared to phenotypes in the observed BC. This procedure revealed that the overlap of the diurnal and lunar QTL at positions 1-M5 and 1-M4 can be expected to result in a significant correlation of lunar and diurnal emergence phase in 90.6% of the cases (Fig. 4). However, in only 301 out of the 10,000 iterations was the resulting correlation as strong as or stronger than the one observed in the backcross. Thus the null hypothesis, that the given genetic architecture explains the observed correlation in the backcross, is rejected with a p value of 301/10000 or 0.0301. However, rejection of the null hypothesis is sensitive to the estimated location and effects of the QTLs. If e.g. instead of weighing the contributions of the QTL by their estimated additive effects (a), the proportion of phenotypic variance explained by the QTL (r^2^) is used, the linkage model to explain the observed correlation in the backcross is accepted with p = 0.0844. But clearly, the genetic architecture as identified in our QTL analysis does not necessarily explain all of the correlation of phenotypes in the backcross and additional loci or other factors influencing the correlation must be considered.

### Genetic differentiation of the linkage groups in field samples

A previous study found genome wide differentiation of *C. marinus* populations as measured with AFLPs to be high at the level of geographically isolated regions (650–1150 km) and low but significant within regions (2–20 km) [20]. When the subset of AFLP markers that is also present on the linkage map presented here (34 markers) is broken down to the level of linkage groups, there is similarly strong differentiation between regions, i.e. on large geographic scales, for all linkage groups (Table 3). Within regions, on a 2 to 20 km scale, there is low but significant differentiation only for linkage group 1, which carries three of the four detected QTLs, while the other two linkage groups do not show significant differentiation (Table 3). This indicates that the timing QTLs could play a role in genetic differentiation between populations of *C. marinus*.

**Table 3:** Genetic differentiation of the linkage groups (LG) in samples from five regions (R) and ten subpopulations (S) all over Europe.

LG	N^a^	F_ST_	p	F_RT_	p	F_SR_	p
1	13	0.262	<10^−5^	0.244	<10^−5^	0.025	0.03
2	13	0.266	<10^−5^	0.259	<10^−5^	0.009	0.28
3	8	0.255	<10^−5^	0.260	<10^−5^	−0.007	0.61

a number of AFLP markers mapped to respective linkage group.

## Discussion

### The candidate genes

The overlap of the QTL with some of the mapped genes produces a number of genes as candidates for being involved in the differences in phase of the diurnal and the lunar rhythm. Candidate genes could be involved in the circadian or lunar oscillators, their light-mediated input pathways, or their output pathways to the developmental mechanisms controlling adult emergence.

For the diurnal candidate genes the place of action can be narrowed down further based on previous experiments which assessed the phase of the circadian oscillators of the *Por* and *Jean* strains. The phase of a biological oscillator can be assayed in a so-called *phase-response experiment*. To test the phase response of the circadian clock, several experimental groups of individuals are released from a 24 hour light-dark regime into constant darkness, so that the circadian oscillator and the observed rhythms that are controlled by it are now free-running without external stimuli. Then for each experimental group the darkness is interrupted by a short light period (several minutes) at different times of the first 24 hours of constant darkness. Depending on the time of light administration, light will be “expected” or “not expected” by the free-running circadian oscillator. As a consequence of the integration of the light stimulus, the phase of the oscillator and thereby the observed rhythm may shift up to several hours to either direction. Since the size and direction of the shift directly depend on the phase of the oscillator, it is called the *phase response*. Conversely, the phase response constitutes an indirect measure of the phase of the oscillator. For the *Por* and *Jean* strains of *C. marinus*, circadian phase responses have been previously measured [21]. The phase response is small and very similar for the *Por* and *Jean* strains, suggesting that if the circadian oscillators differ in phase at all, they certainly differ by less than the observed 4.6 hours difference in diurnal emergence times [21]. This suggests that at maximum one of the genes that account for the differences in diurnal emergence time should act in the circadian oscillator or in the input pathways that synchronise that oscillator. The other one – or maybe even both – should act on the output pathways of the circadian clock.

For the lunar rhythm there is no experimental evidence that would allow to assess if the strains differ in the phase of the underlying circalunar oscillator. Thus it is unknown whether the factors controlling the lunar timing differences are in the lunar oscillator itself, in the light input pathways or in the output pathways.

One candidate gene is *ciliary opsin 2* (*cOps2*), which was mapped to locus 1-M6, within the 1 LOD confidence interval of the diurnal QTL at 1-M5. This means that it lies within the interval where a certain statistical parameter for QTL detection, the so-called *log of odds* (LOD), is within the value 1 from its maximum. At the same time, cOps2 is still within the 2 LOD confidence interval of the lunar QTL at 1-M4. Being a putative light receptor in ciliary photoreceptor cells [22], [23], *cOps2* would most likely be involved in synchronisation (entrainment) of rhythms by light stimuli. In this case it would act in the light input pathways of the oscillators and could influence the phases at which they cycle. Thus we regard *cOps2* as a plausible candidate to explain the lunar timing differences. However, based on the above considerations, on the phase of the circadian oscillator, it is questionable whether *cOps2* is involved in the diurnal timing differences between the *Por* and *Jean* strains. As additionally, the overlapping diurnal and lunar QTLs may contain two independent genes, one for each rhythm, diurnal emergence time could well be controlled by a different but closely linked gene.

The two *timeless* homologues *tim2* (also *timeout* in *D. melanogaster*) and a newly identified *tim3* were both mapped to the second diurnal QTL at locus 1-M16. As recently established for *D. melanogaster*, *tim2* is essential for chromosome integrity but is also involved in light entrainment of the circadian clock [24]. Therefore, just as for *cOps2*, it suffers from the problem of reconciling its putative function in the light input pathways of the circadian oscillator with the *C. marinus* phase response data. *Tim3* is not present in *D. melanogaster*; in fact we can only identify *tim3* homologues in *Culex quinquefasciatus* and *Aedes aegypti* (where they are annotated as *tim2*, although in both species clearly another gene is the true *tim2* ortholog; see Fig. S1). The function of *tim3* is unknown. *Tim1* (*timeless* in *D. melanogaster*) in turn is not only involved in photosensitivity of the circadian clock, but also has a major role in the core clock [25], [26]. In the light of that it seems likely that *tim3* may have a clock function as well.

For the second lunar QTL at locus 2-M10 there is no mapped candidate gene on the male-informative map. But the *cryptochrome1* (*cry1*) homologue at locus 2-F15 of the female-informative map might fall within the ranges of this QTL. In *D. melanogaster* there is only one *cry* gene, which is the orthologue of *cry1* and is assumed to be the major light receptor for entrainment of the circadian clock [27], [28]. In the coral *A. millepora* expression levels of a *cry* homologue were found to differ between full moon and new moon nights [7]. It is unclear whether this change in expression is endogenously controlled by a lunar clock or imposed exogenously by the different levels of nightly illumination. In the latter case, the *cry* expression level might represent a signal indicating nocturnal light. The circadian clock of *D. melanogaster* was shown to respond to dim night-time illumination (“moonlight”) as well [29]. The response was shown to be independent of the *D. melanogaster cry* gene, though, as *cry* deficient mutants showed the same response as wild-type flies [29]. But as *D. melanogaster* lacks any obvious lunar rhythm, this finding does not necessarily rule out a role of *cry1* in the lunar clock.

The absence of overlap with any QTL indicates that the transcription factors *period, cycle, clock, timeless, cryptochrome2* and *vrille*, or the splicing factor *lark*, or the kinases *mck1* and *ck1*, or the light receptors *cOps1* and *rOps2* do not play a major role in determining the differences in the phases of the diurnal and of the lunar rhythm in *C. marinus*. However, the genes *cry2*, *mck1* and *rOps2* lie within the genomic region that would control the differences between a lunar vs. a semi-lunar rythm, if a genetic basis to this phenomenon were to be confirmed.

### Genetic architecture and the diurnal-lunar correlation

The finding of two major QTL for both the phases of the diurnal and the lunar rhythm are consistent with previous analysis of the phenotype distributions [12]. While the two identified lunar loci were found to be independent, the diurnal loci are linked to a certain degree. The level of linkage (32.7 cM) is difficult to detect, though, given that there were only 67 individuals with known diurnal emergence time in the backcross. Additionally, the additive effects of the two major diurnal loci explain only about 85% of the difference between the F_1_ and the Jean strain. The remaining 15% might be explained by one or several minor effect loci which could be unlinked from the major loci and counteract the partial linkage of the two major factors.

The distribution of diurnal and lunar QTL along the linkage groups underscores the genetic basis of the correlation of the two traits as reported previously [12]. The confidence intervals of the diurnal QTL at 1-M5 and the neighbouring lunar QTL at 1-M4 overlap to a large degree. It cannot be determined whether there are two genes in this region, affecting diurnal or lunar rhythm respectively, or only a single gene affecting both traits. A decision will probably require identification and functional characterisation of the gene(s). In either case this results in a correlation of diurnal and lunar phenotypes, which is confirmed by a re-sampling procedure based on the genetic architecture revealed by QTL mapping (Fig. 4). However, the expected correlation is considerably weaker than the observed correlation in the BC family. Possibly, in each of the parental strains the timing genes have several alleles with slightly differing effects. As a consequence, the phenotype distribution of the parental strains and the F_1_ – which are based on several families – would reflect a higher genetic heterogeneity than the single BC family. This might explain why the expected BC distribution, which is derived from the F_1_ and Jean distributions, is more dispersed than the observed BC distribution. Additionally, there may be minor effect loci beyond the detection limit of our crossing experiment for both diurnal and lunar emergence time, and these may be linked to detected or undetected loci for the respective other trait. Slightly elevated LOD scores and r^2^ values (Fig. 3) suggest that there may be additional lunar and diurnal loci between 1-M12 and 1-M15, as well as additional diurnal loci all along linkage group 2. However, these are not significant in the present cross.

The lunar and diurnal rhythms of *C. marinu*s are both adapted to suitable occasions that depend on the tidal regimes and thus change in a highly correlated fashion when moving from one location to the other along the coast. It has been suggested previously that in case diurnal and lunar timing are affected by non-overlapping sets of genes the high predictability of adaptive combinations of lunar and diurnal timing has selected for genetic linkage of the respective lunar and diurnal timing genes [12]. The low overall synteny of the *C. marinus*, *A. gambiae* and *D. melanogaster* chromosomes, as suggested by Table S4, indicates that during the time of independent evolution of these species, their chromosomes have undergone vast re-arrangements, which certainly had the potential of producing genetic linkage between previously unlinked timing genes. If additional crossing experiments with other *C. marinus* strains should reveal the same distribution of QTLs for diurnal and lunar timing, linkage group 1 could prove to “trap” most adaptive timing loci, as it harbours the QTL that account for 85% of the diurnal difference and about 60% of the lunar difference between the two strains.

### Putative non-recombining regions, sex and timing

Light microscopy of *C. marinus* polytene chromosomes revealed that there are two large non-pairing regions [17]. These regions are located in the middle of chromosome II and at the end of chromosome III and were suggested to represent inversion loops. If they are inversions, there should be no recombination within them, so that they should translate into large groups of completely linked markers on the linkage map. Such groups of linked markers exist (see Table S1 and S2), but their numbers and locations vary between the male and female informative maps and are not consistent with the non-pairing regions in the polytene chromosomes. Thus, it is not possible to match linkage groups and chromosomes based on these supposed inversions.

Strikingly, the regions of non-pairing in the polytene chromosomes were observed in all specimens of *C. marinus* that were ever examined (80 individuals in publication [17]; about 20 individuals in own unpublished experiments). In the case of an inversion system homozygotes should occur, so that the regions of non-pairing should be absent from a number of individuals. As homozygotes are not observed, they might be lethal or avoided by recognition mechanisms during fertilisation. Alternatively, the regions of non-pairing might not represent inversions, but may be due to unknown other reasons. In any case, these regions might play a role in the genetic linkage of the timing traits, e.g. by suppressing recombination in chromosome regions that contain the timing genes. As a next step, it could be tested if the chromosomes containing the non-pairing regions are identical with those containing the diurnal and lunar QTLs. Sequence information of the anchor loci from this study (GenBank Accessions JQ011249 to JQ011276) could be used to make probes for fluorescent in situ hybridisation (FISH) on the polytene chromosomes.

The FISH experiment could at the same time resolve if the sex determining locus is associated with one of the putative inversion loops, as was found to be the case in a number of *Chironomus* species [18]. The sex determining locus was mapped to the middle of linkage group 1, indicating that it could be near the centromere of the corresponding chromosome. Notably, the sex determining locus is on the same chromosome as three of the four timing QTL and very close to the *period* and *vrille* genes. This resembles the situation of other insects in which the *period* locus has been found on the sex chromosome [30], [31]. In *C. marinus* the diurnal emergence times of males and females generally differ by about 60 minutes [11]. The proximity of the sex locus and certain clock genes suggests that these genes might be directly responsible for the difference in emergence times. It would be interesting to find the genetic basis to the phenomenon and to test whether it was shaped by sex specific selection. To this end, *period* and *vrille* as well as other genes around the sex determining locus should be analysed for sex specific alleles.

### Evolution of the timing traits

*C. marinus* is restricted to the rocky intertidal and this habitat is not continuously distributed along the European coastline. There are stretches of predominantly rocky coast which are separated from each other by long sandy beaches. A previous study has established that between stretches of rocky coast, genetic differentiation due to geographic isolation is high and that this likely facilitated the evolution of timing adaptations [20]. In contrast, within stretches of rocky coast genetic differentiation was found to be low. Thus, along rocky coasts, where differences in tidal regime are huge on small geographic scales, it is likely that timing adaptations evolved in the face of gene flow, due to strong local selection pressure. This could provide the basis for ecological speciation without geographic isolation. We would expect that gene flow between populations generally attenuates differentiation of the local genomes, but that local selection for loci controlling timing adaptations prevents introgression in these regions of the genome. As a result, the genomic regions that contribute to timing adaptations should be more differentiated between adjacent populations than the rest of the genome. Additionally assuming that all populations achieve local adaptation by the same loci, linkage group 1, which carries 3 of 4 QTL, should be more differentiated between populations than the other linkage groups. Indeed, AMOVA analysis of a limited number of AFLP loci indicates that within stretches of rocky coast on a scale of 2 to 20 km, linkage group 1 – which carries three of the four timing QTL – is significantly differentiated between populations, while linkage groups 2 and 3 are not (Table 3). Future studies with higher marker densities will enable the study of this phenomenon in more detail by assessing the differentiation along the linkage groups.

### Conclusion

Local adaptation in lunar and diurnal emergence times of the marine midge *Clunio marinus* is achieved by two major loci for each trait. The distribution of these loci along the linkage groups underscores non-independent inheritance of the two traits, which can be considered adaptive given the high predictability of good combinations of lunar and diurnal timing in *Clunio's* natural environment. Our results offer candidate genes and a genetic toolkit which may allow identification of the genes underlying the timing adaptations of *C. marinus* in particular and elucidation of the genetic clockwork of lunar rhythmicity in general. Beyond that, fundamental questions in the evolution of life-history traits can be addressed in this system: Can selection shape genome architecture towards genetic linkage of certain traits? Does this involve vast chromosomal rearrangements and/or inversion systems? Can local selection be sufficient to produce adaptation in life history traits without geographic isolation, bearing the potential for ecological speciation? The linkage and QTL maps presented here can serve as a starting point to study these questions in a unique model system – *Clunio marinus*.

## Materials and Methods

### Laboratory stocks and crosses

Laboratory stocks of *C. marinus* from Port-en-Bessin (referred to as Por; Normandie, France) and St. Jean-de-Luz (referred to as Jean; Basque Coast, France) were established from field caught copulae in September 2007 or October 2007 respectively. The laboratory stocks were bred according to Neumann [11]. Temperature in the climate chambers (Snijders Economic Premium) was at 20°C, relative humidity at 50% and the light dark cycle (LD) was 14∶10, with 5000 lux during the day and one hour each of stepwise dusk and dawn (4 steps). Full moon was simulated with an incandescent flashlight bulb (about 1 Lux) switched on all night for four successive nights every 30 days. The newly established strains were kept under laboratory conditions for one generation before starting the crosses; timing adaptations have been stably maintained for more than four years now. Crosses were carried out as single pair matings. We first produced hybrids of the two strains in both directions (Por×Jean, Jean×Por) and then performed backcrosses to the Jean strain in both directions (F_1_×Jean, Jean×F_1_). We chose the largest family “Jean×(Jean×Por)-5” (54 individuals) for the mapping.

### Phenotype scoring

Lunar and diurnal timing phenotypes were recorded for all individuals. The lunar phenotypes (in units of days) are recorded as the day of the artificial moonlight cycle when the individual emerged, “day 1” being the first day with artificial moonlight in the laboratory moonlight cycle of 30 days. The diurnal phenotypes (in units of hours) were recorded in reference to the phase of the light-dark cycle in the respective climate chamber. Following Neumann [11], the middle of the dark phase was defined as “hour 0”, thus making the middle of the light phase “hour 12”. For the parental generation and the F_1_ generation, diurnal emergence times were recorded continuously while performing the crosses and later grouped into 30 minute intervals. For the backcross progeny, diurnal emergence times were recorded by catching all emerged midges in 30 minute intervals.

The timing traits of the parental strains, the F_1_ and the backcross to Jean (BC) have been published previously [12]. Family “Jean×(Jean×Por)-5” is presented with 57 individuals there and also in Fig. 1 of this manuscript, but as 3 males escaped and were not available for DNA extraction, only 54 individuals could be included in the genetic mapping. In the previous publication eight individuals are assigned to the second lunar peak. One of these individuals, which was originally assumed to be emerging late in the second lunar peak based on its lunar and diurnal phenotypes, was now identified to be emerging early in the first lunar peak based on its genotype at the loci controlling the lunar phase of emergence. Thus, the number of individuals in the peaks changes to 7 in the second peak and 50 in the first peak (of which 3 could not be included in the mapping as they escaped). Inclusion of this individual in the first peak also affects the correlation of lunar and diurnal phenotypes; while the correlation was originally reported with ρ = 0.65 and P<8.5×10^−9^ for the full BC, it is now ρ = 0.55 and P<3.5×10^−6^ for the full BC or ρ = 0.61 and P<9.8×10^−6^ for family “Jean×(Jean×Por)-5” alone. To allow inclusion of the seven individuals of the second peak in the QTL mapping for the lunar phase of emergence, their lunar emergence times were matched with those of the individuals in the first peak by subtracting 14 days, i.e. the rounded difference between the mean emergence times in the first and the second peak. To make sure that this procedure does not produce artefacts, QTL analysis was also performed without these seven individuals. This did not affect QTL detection or location.

As recording of diurnal phenotypes was not possible throughout the full emergence period of more than 2 months, 8 of the 54 progeny have imprecise data on their diurnal emergence time. One midge emerging on day 30 and two on day 1 of the lunar cycle emerged sometime later than hour 18.5 in the artificial LD cycle, in the second half of the emergence peak. We set the diurnal phenotype to hour 18.5 for these individuals. Four individuals emerging on day 11 of the lunar cycle emerged sometime before hour 17.5 in the artificial LD cycle, in the early tail of the emergence peak. For these the diurnal phenotype is set to hour 17. To make sure that the assignment of approximated phenotypes to these individuals does not affect the QTL mapping, we also assessed datasets in which these individuals were assigned to earlier or later phenotype classes. This slightly changed the LOD scores and estimates of r^2^ and a, but did not affect the number and location of QTL detected. For one individual diurnal emergence time is unknown.

Due to malfunctioning thermostats, the different generations – parental, F1 and BC – were reared at different temperatures. This affected the phenotype values in Table 2 and Figure 2 (for detailed discussion see [12]). It did not affect the QTL mapping or the genetic correlation of lunar and diurnal timing, as both are based solely on comparisons within the backcross family, which was reared at a consistent temperature of 20°C.

### DNA extraction and amplification

Genomic DNA of all BC progeny, the hybrid F_1_ individual and the Jean strain BC parent, as well as the parents of the F_1_ hybrid, was extracted using a salting out method [32]. To allow for amplification of all desired markers, DNA of all specimens was amplified using the REPLI-g whole genome amplification kit (Qiagen) according to the manufacturer's instructions. Comparative tests of original vs. amplified DNA confirmed that whole genome amplification does not affect patterns in the genetic markers employed. As *C. marinus* adults lack a digestive tract, there is also no risk of amplifying contaminating DNA from gut contents.

### AFLP markers

Amplified fragment length polymorphisms (AFLPs) were obtained following the original protocol by Vos et al. [33] with a few modifications. 200 ng of amplified genomic DNA were digested with EcoRI and MseI (both NEB; 2 h at 37°C) and enzymes were subsequently inactivated (15 min, 65°C). Then EcoRI and MseI adaptors were ligated to the fragments (2 h, 16°C) using T4-DNA-Ligase (NEB). The ligations were diluted 1∶10 and 2 µl of the diluted ligation was used in the pre-amplification. Pre-amplification PCR (20 cycles: 30 s at 94°C, 1 min at 56°C, 1 min at 72°C) was done with primers not containing any selective bases (EcoRI: 5′-GACTGCGTACCAATTC; MseI: 5′-GATGAGTCCTGAGTAA). PCR products were diluted 1∶50 and 2 µl of the dilution were used as a template for the selective amplification (12 cycles: 30 s at 94°C, 30 s at 65°C minus 0.7°C/cycle, 1 min at 72°C; followed by 23 cycles: 30 s at 94°C, 1 min at 56°C, 1 min at 72°C). Primer combinations for selective amplification are given in Table S5. PCR products were again diluted 1∶5 and run on a LICOR 4300 DNA Analyzer. AFLP gels were calibrated and de-smiled using SAGA Generation 2 software from LI-COR, but scored manually for greater confidence. Because AFLP markers are dominant, the only AFLPs that can be unequivocally placed on the map are those for which homozygous recessive genotypes (i.e. absence of a band) and heterozygotes (presence of a band) are segregating 1∶1 in the backcross. These markers are necessarily either male or female informative, but never informative in both directions. Therefore, two independent maps were established.

### Microsatellite markers

Ten microsatellite loci were amplified and scored according to Kaiser & Heckel [34]. Two of these loci – MS13 and MS33 – were not informative in the crossing family and can thus not be found on the linkage map. Six loci were only informative in either male or female direction.

### Anchor loci

In order connect the male and the female informative maps, several AFLP bands from the male informative map as well as a few conserved genes from a cDNA library were chosen as anchor loci to be mapped on both maps. An overview of anchors is found in Table S3. The respective AFLP bands were cut from the polyacrylamide gels and extracted from the gel matrix by incubating the gel piece in 30 µl TE at 60°C for 4 hours. Then the gel piece was subjected to 3 freeze-thaw cycles. The bands were re-amplified by PCR using the specific selective primers, cut from an agarose gel and extracted using the Zymoclean Gel DNA Recovery Kit (Zymo Research), inserted into the pCR2.1-TOPO vector with the TOPO TA Cloning Kit (Invitrogen) and transformed into *E. coli* TOP 10 chemically competent cells. The insert was sequenced on an ABI PRISM 3730xl DNA Analyser (Applied Biosystems) from both ends using M13 primers. In two cases several different sequences were obtained. We chose the one with the length expected from the AFLP gel and verified the identity of the sequence and the AFLP band by scoring a male informative polymorphism from the sequence and checking if it showed the same pattern of inheritance as the original AFLP band. Based on the sequence of the AFLP band we did genome walking usually in one, for some cases in both directions, using the DNA Walking SpeedUp kit (Seegene). The obtained PCR products were cut from an agarose gel, extracted using the Zymoclean Gel DNA Recovery Kit (Zymo Research) and then sequenced directly on an ABI PRISM 3730xl DNA Analyser (Applied Biosystems). The sequences were deposited in GenBank (GenBank Accessions JQ011249 to JQ011260). Sequences were checked for the presence of coding sequences (CDS) by comparing them to the nr database at NCBI using the tBLASTx algorithm (Table S3). Finally, the sequence was amplified from the F_1_ individual and the BC parent, sequenced from both ends and searched for informative polymorphisms by visually evaluating the chromatograms. Single nucleotide polymorphisms (SNPs) and short indels (1–3 bp) were scored by amplifiying, sequencing and visually evaluating the sequence for all BC progeny. For longer indels, the length differences were scored on polyacrylamide gels or even on agarose gels.

### Candidate genes

As candidate genes, we cloned and mapped *C. marinus* homologues of several genes that are known to be involved in the circadian clock or in light perception in other organisms. An overview of the known or assumed functions of these genes is given in Table 1. Sequences were either obtained by degenerate PCR or from a normalised cDNA library of all life stages of *C. marinus*. An overview of sequences and how they were obtained is given in Table S6. The partial sequences used for identifying and mapping these homologues are deposited in GenBank (GenBank Accessions JQ011261 to JQ011276). For the gene families of *cOpsins*, *cryptochromes* and *timeless* genes, the identity of orthologs and paralogs was determined by phylogenetic analysis (Figures S1, S2 and S3). Detailed descriptions of the cDNA library and of the genes obtained by degenerate PCR will be presented in separate publications that are in preparation. Using PCR primers based on the partial cDNA sequences, we amplified the respective genomic region from the genomic DNA of the F_1_ individual and the BC parent, sequenced it from both ends and searched for informative polymorphisms by visually evaluating the chromatogrammes. Single nucleotide polymorphisms (SNPs) and short indels (1–3 bp) were then scored by amplifiying, sequencing and visually evaluating the sequence for all BC progeny.

### Linkage map construction

The BC family consisted of 54 individuals genotyped for 344 markers (312 AFLP markers; 8 microsatellite markers; 24 anchor and gene loci). 189 markers were male informative, 162 were female informative. As with the given number of markers the *C. marinus* genome is densely covered, it was easily possible to recognize the gametic phase of the markers and invert scoring patterns for dominant markers in opposition. Markers with identical patterns were grouped into marker groups (Table S1 and S2). The sets of male and female informative marker groups in the same phase were then used to produce two separate linkage maps using Mapmaker 3.0 [35]. The input files are provided as dataset S1 and S2. Linkage groups were established using the “group” command with the Kosambi mapping function. The two-point linkage criteria were a minimum LOD score of 3.0 and a maximum distance between markers of 20 cM. The map order was estimated and refined using the “compare” and “ripple” commands. In case a double recombination event was only supported by a single marker, this was considered a PCR artefact and the marker was removed from the dataset. This might have resulted in an overly conservative approach, underestimating the true length of the map.

### Estimation of genome length and map coverage

Genome length L was estimated by multiplying the length of each linkage group by the factor (m+1)/(m−1), where m is the number of marker groups on each linkage group (method 4 in [36]). Under the assumption that markers are randomly distributed, map coverage was estimated by c = 1−e^−2dn/L^, c being the fraction of the genome being within d cM of a marker, L the genome length and n the number of markers [37].

### Flow cytometry

To get an estimate of the size of the *C. marinus* genome, nuclei were extracted and stained using the CyStain® UV Precise T kit (Partec) and run on a Partec PA-II flow cytometer. Nuclei of *Drosophila elegans* and *Drosophila subobscura* served as a reference. The *Drosophila* reference individuals were obtained from the stocks of Dr Andrew Davis at the Max Planck Institute for Chemical Ecology (Jena, Germany).

### Genome structure comparison with other dipterans

In order to get an idea of the level of synteny between the *C. marinus* genome and other dipteran genomes, we tried to find the homologous sequences in *Drosophila melanogaster* and *Anopheles gambiae* for all gene markers on our map. The respective sequences from *C. marinus* were blasted against the *Drosophila* and *Anopheles* proteins in the nr database at NCBI in a blastx search. If there was a single best hit, separated from the second best hit by a substantial decrease in the E-value, the corresponding chromosome arm locations in *Drosophila melanogaster* and *Anopheles gambiae* were retrieved (Table S4).

### Composite interval mapping

The QTL for the phase of the diurnal rhythm and the phase of the lunar rhythm were mapped in the BC progeny using a composite interval mapping (CIM) approach [38] as implemented in Windows QTL cartographer 2.5 [39]. CIM combines interval mapping as developed by Lander *et al.* [35] with multiple regression to test for the presence of QTL within a marker interval, while using other markers as co-factors to account for QTL outside the test interval. CIM was run with the standard model and a forward regression method. The number of cofactors in the multiple regression was 5. The size of the exclusion window around the test interval was optimised to achieve a compromise between map resolution and estimates of the additive effects. With smaller exclusion windows the QTL were generally narrower, but the sum of their estimated additive effects tended to be further from the observed difference between the F_1_ and the Jean strain. Eventually, the exclusion window was 5 cM for the diurnal QTL map and 8 cM for the lunar QTL map. The likelihood ratio test statistic was calculated every 0.5 cM. The QTL significance thresholds were estimated by permutation tests (1000 permutations) to obtain a significance level of 0.05. The input file is provided as dataset S3.

### Estimation of QTL effects

Additive effects (a) and the proportion of phenotypic variance explained (r^2^) were estimated using Windows QTL Cartographer 2.5 [39]. The proportion of variance explained by a QTL is estimated by r2 = (s_0_^2^−s_A_^2^)/s^2^, where s^2^ is the trait variance, s_0_^2^ is the sample variance of the residuals under the null model and s_A_^2^ is the variance of the residuals under the alternative model [40]. Estimates of a are obtained through a maximum likelihood based expectation/conditional maximisation algorithm [41].

### Estimation of the correlation of lunar and diurnal phase of emergence

With the knowledge of the map location and the additive effects of timing loci coming from this study, we refined the genetic model used in a previous study [12], in order to test if the given genetic architecture can be expected to result in a correlation of the timing traits and to assess the strength of the expected correlation. We used a re-sampling procedure in which the expected phenotype distribution in the major peak of a backcross family was obtained from the observed phenotype distribution in the F_1_ and in the strain of the BC parent (Jean strain). The number of individuals was 63, equalling the number of individuals with known diurnal emergence time in the major peak of the full backcross of the previous study. For each individual we obtained its genetic composition by randomly determining if for the major diurnal locus it received a Por-type allele or a Jean-type allele from the F_1_ individual. Then the alleles at the other loci were determined depending on the allele at the first locus and on the levels of the observed genetic linkage between the loci. For example, if in the 54 individuals of our BC family there were 17 recombination events between two loci, we assumed that in 17 of 54 cases these two loci would have inherited a Por allele for one locus and a Jean allele for the other locus from the F_1_ individual, while in 37 of 54 cases the two loci would have received two alleles of the same parental strain from the F_1_ individual. In the model the two diurnal loci on linkage group 1 are separated by 17 recombination events, and the lunar locus on linkage group 1 is separated by 2 recombination events from the first diurnal locus. For unlinked loci, the allele coming from the F_1_ parent was randomly assigned.

Based on the genetic composition of all loci, the phenotype was determined in the following way: If an individual had only Jean alleles at all loci for a given trait, the phenotype was obtained by sampling from the phenotype distribution of the Jean strain. If an individual had a hybrid composition of alleles at all loci for a given trait, i.e. the same composition as an F_1_ individual, the phenotype was obtained by sampling from the phenotype distribution of the F_1_. If the genetic composition was Jean-like for some loci and F_1_-like for others, a corresponding phenotype was sampled for each locus and the composite phenotype resulted from weighting the sampled phenotypes according to the estimated additive effects (a) of the corresponding loci. The phenotypes of the sample BC family were grouped according to the categories in the observed BC (days for the lunar rhythm; 30 min intervals for the diurnal rhythm). Then the correlation of diurnal and lunar phenotypes was assessed by Pearson's product moment correlation. This procedure was repeated 10.000 times and p values for the correlations were recorded. The fraction of significant p values was determined in order to assess if a significant correlation of the phenotypes can generally be expected. Finally, the fraction of p values smaller or equal to the p value of the observed BC was used to assess the null hypothesis that the given genetic architecture can explain the level of correlation observed in the BC family.

### Genetic differentiation of the linkage groups in field samples

The data used to assess genetic differentiation of the linkage groups in field samples was retrieved from a previous study, which assessed genetic differentiation at two rather different geographic levels [20]. Samples came from five regions all over Europe (Vigo/Spain; Basque Coast/France; Normandie/France; Helgoland/Germany; Bergen/Norway) which are 650 to 1150 km apart and generally separated by effective geographic barriers. Within regions usually several local populations were sampled, at a scale of 2 to 20 km distance. As the population study involved a much lower number of AFLP primer combinations, only 34 of the AFLP markers from the linkage map are also present in the population study. The identity of bands was assessed by visually comparing the two AFLP gel pictures. As the degree of polymorphism in AFLPs is extremely low in *C. marinus*, the AFLP gels highly resemble each other and landmark bands are readily available. Homoplasies cannot be excluded, but are unlikely given the fact that the density of AFLP markers on the gels is low. The data for the 34 AFLP markers with known map location were retrieved from the population study and grouped according to the three linkage groups. Then differentiation of the three linkage groups was assessed independently in an analysis of molecular variance (AMOVA) as implemented in Arlequin ver 3.5.1 [42].

## Supporting Information

Figure S1 Neighbour Joining tree of dipteran timeless genes including bootstrap values (10.000 replications).
(DOC)

Figure S2 Neighbour Joining tree of insect cryptochrome genes including bootstrap values (10.000 replications).
(DOC)

Figure S3 Neighbour Joining tree of insect cOpsin genes including bootstrap values (10.000 replications).
(DOC)

Table S1 Marker groups on the male informative map.
(DOC)

Table S2 Marker groups on the female informative map.
(DOC)

Table S3 Anchor loci.
(DOC)

Table S4 Genomic location of selected gene loci in *C. marinus*, *D. melanogaster* and *A. gambiae*.
(DOC)

Table S5 AFLP primer combinations.
(DOC)

Table S6 Clock genes and light receptors.
(DOC)

Dataset S1 Mapmaker input file for male informative marker groups.
(TXT)

Dataset S2 Mapmaker input file for female informative marker groups.
(TXT)

Dataset S3 WinQTL input file.
(TXT)
