# Supplementary material for: Genetic Architecture of Local Adaptation in Lunar and Diurnal Emergence Times of the Marine Midge Clunio marinus (Chironomidae, Diptera)
Source: PLoS One. 2012 Feb 22;7(2):e32092. doi: 10.1371/journal.pone.0032092 (PMC3285202; doi:10.1371/journal.pone.0032092)
Supplement: Figure S2 — Neighbour Joining tree of insect cryptochrome genes including bootstrap values (10.000 replications). (DOC) [file pone.0032092.s002.doc]

**FigureS2**  Neighbour Joining tree of insect cryptochrome genes including bootstrap values (10.000 replications).
