# Supplementary material for: Genetic Architecture of Local Adaptation in Lunar and Diurnal Emergence Times of the Marine Midge Clunio marinus (Chironomidae, Diptera)
Source: PLoS One. 2012 Feb 22;7(2):e32092. doi: 10.1371/journal.pone.0032092 (PMC3285202; doi:10.1371/journal.pone.0032092)
Supplement: Table S1 — Marker groups on the male informative map. (DOC) [file pone.0032092.s004.doc]

**Table S1**

Marker groups on the male informative map

| **Linkage group** | **Marker group** | **Genes** | **Micro-satellites** | **AFLP markers** |
| --- | --- | --- | --- | --- |
|  |  |  |  |  |
| 1 | 1-M1 | - | - | CT-ACT-242 |
|  | 1-M2 | - | - | AG-GCG-195 |
|  | 1-M3 | **Structural maintenance of chromosomes protein 4 (SMC4; GA-CGC-4xx)** | MS15 | AA-ATG-311, AA-CGA-156, AA-CGA-309, AC-ACT-298, AC-TCT-106. AG-ACA-270, AT-GTA-182, CT-TAC-46, GG-AAC-362, TA-ACT-235, TA-CGC-152, TA-GTA-175, TC-CGC-156, TG-ACA-189, TT-AAC-277, TT-ACA-214, TT-ATG-573 |
|  | 1-M4 | - | - | TG-ATG-365 |
|  | 1- M5 | - | - | CT-ACT-209 |
|  | 1-M6 | **Ciliary Opsin 2 (cOps2), ribosomal protein L15 (RP L15)** | MS30 | AA-AGG-228, AA-AGG-55, AC-CAT-80, AG-AAC-145, AT-ACG-179, AT-ATG-199, AT-TAC-158, GA-CGC-287, GG-ATG-96, GT-ACT-217 |
|  | 1-M7 | - | - | AG-AAC-83, CA-ACT-167, TT-ACA-192 |
|  | 1-M8 | - | - | AC-CAT-66, AG-CAT-279, AT-AAG-139, GA-ATG-228, GT-AAC-179 |
|  | 1-M9 | - | - | GG-ACG-105 |
|  | 1-M10 | **Ciliary Opsin 1 (cOps1)** | - | AC-ACA-110, AG-AAC-280, AT-GTA-200, GC-AAC-145, GG-ACT-98, GG-ACT-171 |
|  | 1-M11 | - | - | AG-ATG-88, AT-AAG-148, AT-ACG-149 |
|  | 1-M12 | - | - | AG-CAT-350, AT-GTA-118, AT-TAC-141 |
|  | 1-M13 | Similar to tyrosine kinase “shark” (TG-ACA-211) | - | TG-ACA-211 |
|  | 1-M14 | - | - | GT-ATG-132 |
|  | 1-M15 | **MDH (GG-ATG-270)** | - | AT-TAC-190, GA-ACA-139, GG-ATG-270 |
|  | 1-M16 | **Timeless3 (Tim3)** | - | AA-ACT-360, AT-AAC-187, AT-ATG-295, AT-ATG-296, TA-CGC-240, TA-TAC-182 |
|  | 1-M17 | - | - | AA-ATG-174, GT-AAC-112, GT-AAC-209 |
|  | 1-M18 | - | - | TT-ACT-134 |
|  |  |  |  |  |
| 2 | 2-M1 | - | - | AA-ACT-180 |
|  | 2-M2 | - | - | GT-AAC-259 |
|  | 2-M3 | Poly-A-Polymerase (TG-AAC-288) | - | AA-ATG-272, AG-ATG-106, CT-ATG-569, GG-ACA-210, GT-ACT-278, TG-AAC-288 |
|  | 2-M4 | - | - | AT-ACA-160, GA-ACC-142 |
|  | 2-M5 | - | - | AC-ACT-260 |
|  | 2-M6 | - | - | AG-ACG-114 |
|  | 2-M7 | - | - | TT-ATG-72 |
|  | 2-M8 | ? (AG-ACA-351) | - | AG-ACA-351, TG-AAC-95 |
|  | 2-M9 | - | **MS23** | - |
|  | 2-M10 | - | - | GT-AAC-145, TT-AAC-326 |
|  | 2-M11 | - | - | AG-TCT-142, CT-ATG-356, CTT-ACG-355, TT-ATG-354 |
|  | 2-M12 | - | - | TC-AGA-362 |
|  | 2-M13 | - | - | AG-ACA-127 |
|  | 2-M14 | - | - | TA-ACA-310 |
|  | 2-M15 | - | - | TA-AAC-122 |
|  | 2-M16 | - | - | AC-ACT-174, CT-TAC-291, TA-ATG-113 |
|  | 2-M17 | - | - | TG-ACT-170, TG-ACT-174 |
|  | 2-M18 | - | - | AT-ACG-240, CTT-ACG-67, GA-AAC-295 |
|  | 2-M19 | **Cryptochrome 2 (Cry2; “mammalian type”)** | - | AA-ATG-227, AG-AAG-192, AT-AAG-287, AT-ATG-175, CT-GTA-138, GA-ACA-90, GA-CGC-387, GG-AAC-73 |
|  | 2-M20 | Rhabdomeric Opsin 2 (rOps2), **U6 snRNA-associated Sm-like protein LSm3 (GA-ACT-469)** | - | AA_CGA-335, AG-ACC-115, AT-AAC-260, AT-TAC-167, CT-TAC-143, GA-ACT-469, GC-ACA-547, GG-ACA-293, GT-ACC-86, TA-CAT-146, TG-ACC-154, TG-ACC-241, TT-ACA-104, TT-ACT-248 |
|  | 2-M21 | - | - | AT-ACT-119 |
|  | 2-M22 | - | - | TG-ATG-110 |
|  |  |  |  |  |
| 3 | 3-M1 | - | - | TG-ATG-279 |
|  | 3-M2 | - | - | AT-ATG-147, GA-ATG-262, TA-TAC-266, TG-AAC-234 |
|  | 3-M3 | **Timeless (Tim), Globin, (Lyosomal?) Lipase** | MS7, **MS32** | AA-AGG-209, AA-ATG-98, AC-AAC-331, AC-ACA-96, AC-ACA-98, AC-ATG-134, AG-ACC-164, AG-ACC-282, AG- TCT-107, AT-ACT-240, AT-ATG-188, AT-ATG-92, AT-GTA-102, AT-GTA-139, AT-TAC-68, GA-ACA-293, GA-ACT-238, GG-ACA-111, GG-ACA-75, GT-ACT-199, GT-ATG-151, GT-ATG-318, TA-ACT-338, TA-ATG-101, TA-TAC-65, TC-AGA-67, TT-ACT-216 |
|  | 3-M4 | - | - | AC-CAT-231, GT-ACA-137, TC-AGA-202 |
|  | 3-M5 | **Clock (Clk), unknown gene with myosin binding subunit (AG-ACA-476)** | - | AA-ACT-197, AC-AAC-295, AC-ACT-77, AG-ACA-476, AT-ACT-143, GT-ACT-117 |
|  | 3-M6 | - | - | GC-ACA-94 |
|  | 3-M7 | - | - | TC-AGA-117 |
|  | 3-M8 | - | - | AT-ATG-315 |
|  | 3-M9 | - | - | TA-AAC-109 |
|  | 3-M10 | **Titin (AC-ACT-546)** | - | AC-ACT-186, AT-ACT-546, GC-AAC-88 |
|  | 3-M11 | - | - | TA-CAT-74AG-CAT-83 |

Bold names indicates that gene marker has male and female informative polymorphisms and could therefore be placed on both maps.
