# Supplementary material for: Genetic Architecture of Local Adaptation in Lunar and Diurnal Emergence Times of the Marine Midge Clunio marinus (Chironomidae, Diptera)
Source: PLoS One. 2012 Feb 22;7(2):e32092. doi: 10.1371/journal.pone.0032092 (PMC3285202; doi:10.1371/journal.pone.0032092)
Supplement: Table S2 — Marker groups on the female informative map. (DOC) [file pone.0032092.s005.doc]

**Table S2** Marker groups on the female informative map

| **Linkage group** | **Farker group** | **Genes** | **Micro-satellites** | **AFLP markers** |
| --- | --- | --- | --- | --- |
| 1 | 1-F1 | **Structural maintenance of chromosomes protein 4 (SMC4; GA-CGC-4xx)** | - | AG-AAC-148, AG-TCT-92, AT-ACT-230, GT-ACC-147, GT-ACC-73, TC-AGA-276, TT-AAC-282, TT-ATG-321 |
|  | 1-F2 | - | - | TC-AGA-430 |
|  | 1-F3 | **Ciliary Opsin 2 (cOps2), ribosomal protein L15 (RP L15)** | - | AT-ATG-133 |
|  | 1-F4 | - | - | AA-AGG-301, AC-ATG-607, AC-ATG-85, AG-ACA-186, AT-ATG-115, AT-TAC-162, CT-ACT-447, GA-CGC-150, GT-ACT-245, TA-ATG-258 |
|  | 1- F5 | - | - | TG-AAC-135 |
|  | 1-F6 | - | - | CA-ATG-273, GA-AAC-76 |
|  | 1-F7 | - | - | AA-CGA-115, AT-AAC-136, GA-ACA-234, GG-AGG-94 |
|  | 1-F8 | - | - | AA-ATG-670, AT-CGC-220, TA-ATG-350, TA-GTA-113 |
|  | 1-F9 | **Ciliary Opsin 1 (cOps1)** | - | AA-ATG-392, GA-ACA-118, GG-ACA-312, TA-ACT-205, TG-ATG-241, TT-AAC-217 |
|  | 1-F10 | Period (per) | - | - |
|  | 1-F11 | - | - | AC-ATG-281, TG-CGC-159, TT-ACA-352 |
|  | 1-F12 | - | - | AA-AGG-82, AG-CAT-140, AG-CGC-286 |
|  | 1-F13 | - | - | GG-ACA-99 |
|  | 1-F14 | **MDH (GG-ATG-270)** | - | GA-ATG-153 |
|  | 1-F15 | - | - | GT-ATG-380 |
|  | 1-F16 | Timeless2/Timeout (Tim2), **Timeless3 (Tim3)** | - | AC-AAC-94, AT-AAG-128, AT-GTA-185, CA-GTA-330, CA-TAC-177 |
|  | 1-F17 | - | - | AG-TCT-81, AT-ATG-72, AT-ACA-287, CA-TAC-151 |
|  |  |  |  |  |
| 2 | 2-F1 | Casein Kinase 1a (CK1a) | - | - |
|  | 2-F2 | - | - | GT-CGC-90 |
|  | 2-F3 | - | - | TT-ATG-540 |
|  | 2-F4 | - | - | TA-TCT-163 |
|  | 2-F5 | - | - | TT-AAC-215 |
|  | 2-F6 | - | - | AC-ACT-73 |
|  | 2-F7 | - | - | CTT-AAG-103 |
|  | 2-F8 | - | **MS23** | TA-ACT-130, TA-ACT-229 |
|  | 2-F9 | - | - | TC-AGA-364 |
|  | 2-F10 | - | - | TA-TAC-137 |
|  | 2-F11 | - | - | GG-AGG-136 |
|  | 2-F12 | Ribosomal protein L7 | - | AG-ACA-68, GT-ATG-227 |
|  | 2-F13 | - | - | TA-ACA-236 |
|  | 2-F14 | - | - | AA-ACT-267, AC-TCT-132 |
|  | 2-F15 | Cryptochrome 1 (Cry1; “insect type”) | - | AA-CGA-130, CA-GTA-103, TA-TAC-165, TA-TAC-166 |
|  | 2-F16 | - | - | TA-CAT-303 |
|  | 2-F17 | - | - | TG-ACT-172, TG-ATG-483 |
|  | 2-F18 | - | - | TG-ATG-130 |
|  | 2-F19 | Similar to GH10678-RA (Drosophila grimshawii; TA-ACA-310), **Cryptochrome 2 (Cry2; “mammalian type”), U6 snRNA-associated Sm-like protein LSm3 (GA-ACT-469),**  Ribosomal protein S12 | MS26 | AA-CGA-173, AC-ATG-112, AC-CAT-84, AG-ACA-205, AG-ACA-54, AT-AAG-641, AT-GTA-206, GA-ACA-344, GA-ATG-90, GC-ATG-132, GG-AAC-127, GG-ACT-75, GT-ACA-88, GT-ATG-220, TA-ATG-215, TA-ATG-296, TA-CAT-204, TG-AAC-130, TG-AAC-68, TT-ACA-346 |
|  | 2-F20 | - | - | TA-ACA-234, TA-ACC-201 |
|  |  |  |  |  |
| 3 | 3-F1 | - | **MS32** | AC-AAC-66, AC-ACT-95, AC-TCT-216, AT-GTA-318, CA-ATG-264, CTT-ACG-244, GA-ACA-313, TA-CAT-103 |
|  | 3-F2 | - | - | AC-ACC-90, AC-ATG-188 |
|  | 3-F3 | **Timeless (Tim)** | - | AG-ACA-408 |
|  | 3-F4 | - | - | AT-AAG-313, TA-TAC-125 |
|  | 3-F5 | **Globin, (Lyosomal?) Lipase** | - | - |
|  | 3-F6 | - | MS11 | - |
|  | 3-F7 | - | - | AT-GTA-311 |
|  | 3-F8 | - | MS29 | TA-TCT-148 |
|  | 3-F9 | - | - | AT-ACA-168, AT-GTA-253, CA-GTA-71, CT-TAC-286, TG-CGC-60 |
|  | 3-F10 | - | - | AA-ACT-457, AT-ATG-192, CA-ACT-285, CT-ATG-228, GC-AAC-244, TA-GTA-65, TA-TCT-171 |
|  | 3-F11 | - | - | AT-ACT-88, AT-ACT-94 |
|  | 3-F12 | - | - | GC-AAC-617 |
|  | 3-F13 | **Clock (Clk), unknown gene with myosin binding subunit (AG-ACA-476)** | - | TA-TCT-345 |
|  | 3-F14 | - | - | AC-TCT-181 |
|  | 3-F15 | - | - | AC-ATG-234, AT-AAG-483, GG-AGG-106 |
|  | 3-F16 | **Titin (AC-ACT-546)** | - | TT-ATG-122 |
|  | 3-F17 | - | - | AT-ACG-146 |
|  | 3-F18 | - | - | CTT-ACG-118 |
|  | 3-F19 | - | - | TA-TAC-284 |
|  |  |  |  |  |

Bold names indicates that gene marker has male and female informative polymorphisms and could therefore be placed on both maps.
