# Supplementary material for: Genetic Architecture of Local Adaptation in Lunar and Diurnal Emergence Times of the Marine Midge Clunio marinus (Chironomidae, Diptera)
Source: PLoS One. 2012 Feb 22;7(2):e32092. doi: 10.1371/journal.pone.0032092 (PMC3285202; doi:10.1371/journal.pone.0032092)
Supplement: Table S3 — Anchor loci. (DOC) [file pone.0032092.s006.doc]

**Table S3**

Anchor loci

| **An-chor** | **AFLP** | **Putative gene loci** | **Best blast hit within sequence (bits / e value)** | **Sequence obtained by** | **primers** |
| --- | --- | --- | --- | --- | --- |
| A1 | GA-CGC-400 | Putative transposase  Structural maintenance of chromosomes protein 4 (SMC4) | Putative transposase: Toxorhynchites amboinensis gb|AAL86009.1| put. transposase  97.1 / 2e-20  SMC4:  Aedes aegypti  GENE ID: 5571662 AaeL_AAEL001655 | structural maintenance of chromosomes smc4  104 / 3e-20 | Genome walking from AFLP band | For 5’ – AGGGTTGCTCATTGCTGGTA – 3’  Rev 5’ – CGCTTCTCGAGGCACTCTTT – 3’ |
| A2 | NA | Ribosomal protein L15 | Culex quinquefasciatus  GENE ID: 6040187 CpipJ_CPIJ008262 | 39S ribosomal protein L15, mitochondrial  398 / 4e-109 | CDS from cDNA library | For 5’ – CAACTGTTAAGCATACAACAGAACG – 3’  Rev 5’ – AAGTCCACACGATTCCAGGT – 3’ |
| A3 | TG-ACA-211 | Tyrosine kinase (similar to shark) | Anopheles gambiae GENE ID: 1272509 AgaP_AGAP010710 | 87.8 / 4e-27 | Genome walking from AFLP band | For 5’ – CACCAGAACCAAAGCGATGT – 3’  Rev 5’ – AGCAACCATCGACTTTTCAGA – 3’ |
| A4 ^a^ | GG-ATG-270 | Malat dehydrogenase (Mdh) | Aedes aegypti  GENE ID: 5568365 AaeL_AAEL001091 | malic enzyme  478 / 2e-173 | Genome walking from AFLP band | For 5’ – TCCAACCTTGTCACACTCAGC – 3’  Rev 5’ – TCGTCATATGCTTGGCCTGA – 3’ |
| A5 | TG-AAC-288 | Poly A polymerase | Aedes aegypti  GENE ID: 5566394 AaeL_AAEL005356 | poly a polymerase  353 / 0.0 | Genome walking from AFLP band | For 5’ – TGGAAATGCTGGATGGGAGA – 3’  Rev 5’ – TGTGGTTGGGGAGATGCTTT – 3’ |
| A6 | NA | Ribosomal protein L7 | Culex quinquefasciatus  GENE ID: 6042241 CpipJ_CPIJ010020 | 60S ribosomal protein L7  335 / 3e-90 | CDS from cDNA library | For 5’ – ATGGCACCAACTGCTAAACC – 3’  Rev 5’ – TTCGCTGAAGCAACTCGTTA – 3’ |
| A7 | TA-ACA-310 | Similar to CG15828 | Drosophila grimshawi  GENE ID: 6561417 Dgri\GH10678 | GH10678  80.1 / 5e-25 | Genome walking from anonymous DNA fragment | For 5’ – CATTGGTTGTTTTGCATCCATC – 3’  Rev 5’ – CTCCATTAAATCGTGCATTCG – 3’ |
| A8 | GA-ACT-469 | U6 snRNA-associated Sm-like protein LSm3 (LSM3) | Drosophila pseudoobscura  GENE ID: 6903902 Dpse\GA29224  43.1 / 0.077 | Genome walking from AFLP band | For 5’ – CGAGCAGTTCTTGAGCCAAA – 3’  Rev 5’ – AATGGCTCCTTTACGGGTGT – 3’ |
| A9 | NA | Ribosomal protein S12 | Drosophila mojavensis  GENE ID: 6583875 Dmoj\GI15180 |  186 / 8e-46 | CDS from cDNA library | For 5’ – GAAACAAGCATTCACCGTAACA – 3’  Rev 5’ – AGGCTTTGATGACATGAGGT – 3’ |
| A10 | NA | Globin1  Lipase | Globin:  Drosophila pseudoobscura  GENE ID: 4802325 Dpse\GA21995 |  40.8 / 0.033  Lipase:  Culex quinquefasciatus GENE ID: 6033918 CpipJ_CPIJ002724 | lysosomal acid lipase 60.1 / 1e-06 | Genome Walking from cDNA fragment | For 5’ – GGGCGCAACAATGAATAGAT – 3’  Rev 5’ – TAACAACCCGTGCATCAAAA – 3’ |
| A11 | AG-ACA-476 | Mbs/  unknown gene with myosin binding subunit | Anopheles gambiae  GENE ID: 1277253 AgaP_AGAP006665  97.8 / 7e-21 | Genome walking from AFLP band | For 5’ – TTTGTTGCGCAATCAGAAAG – 3’  Rev 5’ – CCAACAAGTCCATCGACACC – 3’ |
| A12 | AT-ACT-546 | Titin | Anopheles gambiae  GENE ID: 1269678 AgaP_AGAP007556 | AGAP007556-PA  535 / 5e-150 | Genome walking from AFLP band | For 5’ – TTCACCGTTTGCCTTCGTTT – 3’  Rev 5’ – GCACTGAATGGCCTTGAACC – 3’ |

^a^ *Mdh* is probably wrongly annotated as malic enzyme (*Men*) in *A. aegypti*. In *D. melanogaster* and *A. gambiae* the best hit is clearly *Mdh*, followed by *Men* (see Table 2).
