# Supplementary material for: Genetic Architecture of Local Adaptation in Lunar and Diurnal Emergence Times of the Marine Midge Clunio marinus (Chironomidae, Diptera)
Source: PLoS One. 2012 Feb 22;7(2):e32092. doi: 10.1371/journal.pone.0032092 (PMC3285202; doi:10.1371/journal.pone.0032092)
Supplement: Table S4 — Genomic location of selected gene loci in C. marinus, D. melanogaster and A. gambiae. (DOC) [file pone.0032092.s007.doc]

**Table S4**

Genomic location of selected gene loci in *C. marinus*, *D. melanogaster* and *A. gambiae*

| *C. marinus* | | |  | *Drosophila melanogaster* | | |  | *Anopheles gambiae* | | |
| --- | --- | --- | --- | --- | --- | --- | --- | --- | --- | --- |
| **Linkage group** | **Gene** | **Map** |  | **Best blast hit** | **2nd best blast hit** | **Chromosome arm** |  | **Best blast hit** | **2nd best blast hit** | **Chromosome arm** |
| 1 | SMC4 | m, f |  | GENE ID: 35001  glu | gluon  "XCAP-C/SMC4 homolog"  86.3 / 8e-17 | NA (no second hit) | **2L** |  | GENE ID: 1278132 AgaP_AGAP007826  102 / 5e-22 | NA (no second hit) | **3R** |
| 1 | c0ps2 ^a^ | m, f |  | NA | NA | NA |  | GENE ID: 1273518 GPROP12  335 / 8e-90 | GENE ID: 1273519 GPROP11  321 / 2e-85 | **2R** |
| 1 | L15 | m, f |  | GENE ID: 40261 mRpL15 | mitochondrial ribosomal protein L15  371 / 5e-103 | GENE ID: 5740788 CG34382 | CG34382  28.9 / 6.4 | **3L** |  | GENE ID: 1280723 AgaP_AGAP011946 | AGAP011946-PA  386 / 4e-108 | GENE ID: 1274469 AgaP_AGAP004324 | AGAP004324-PA  29.3 / 1.7 | **3L** |
| 1 | cOps1 ^a^ | m, f |  | NA | NA | NA |  | GENE ID: 1273518 GPROP12  254 / 1e-65 | GENE ID: 1273519 GPROP11  240 / 2e-61 | **2R** |
| 1 | Per ^b^ | f |  | GENE ID: 31251  per | period  67.0 / 3e-11 | GENE ID: 42080 Mur89F | Mucin related 89F  33.9 / 0.27 | **X** |  | GENE ID: 3290523 AgaP_AGAP001855  50.1 / 1e-06 | GENE ID: 1274848 AgaP_AGAP005162  32.0 / 0.36 | **2R ?** |
| 1 | Vrille | m, f |  | GENE ID: 33759  vri | vrille  193 / 2e-49 | GENE ID: 45588  Pdp1 | PAR-domain protein 1  64.3 / 1e-10 | **2L** |  | GENE ID: 1278160 AgaP_AGAP007801 | AGAP007801-PA  212 / 4e-53 | GENE ID: 1276989 AgaP_AGAP006376 | AGAP006376-PA  67.4 / 6e-12 | **3R** |
| 1 | Shark | m |  | GENE ID: 44353  shark | Src homology 2, ankyrin repeat, tyrosine kinase  73.2 / 4e-22 | GENE ID: 53425  Alk | CG8250 gene 57.4 / 4e-16 | **2R** |  | GENE ID: 1272509 AgaP_AGAP010710 | 87.8 / 1e-29 | GENE ID: 1271751 AgaP_AGAP000489 |  55.8 / 3e-16 | **3L** |
| 1 | Mdh | m, f |  | GENE ID: 43936  Mdh | CG5889  438 / 1e-157 | GENE ID: 47173  Men | Malic enzyme 381 / 2e-120 | **3R** |  | GENE ID: 1273984 AgaP_AGAP004159 | "similar to Mdh"  478 / 7e-175 | GENE ID: 1272080 AgaP_AGAP000184 |  "similar to Men"  399 / 1e-126 | **2R** |
| 1 | Timeless 2 | f |  | GENE ID: 41615  timeout | CG7855  192 / 2e-49 | GENE ID: 33571  tim | timeless  58.5 / 5e-09 | **3R** |  | GENE ID: 5668064  AgaP_AGAP010787 169 / 8e-43 | GENE ID: 5668288  AgaP_AGAP008288 66.6 / 7e-12 | **3L** |
| 1 | Timeless 3 ^c^ | m, f |  | GENE ID: 41615 timeout | CG7855  334 / 2e-89 | GENE ID: 37924  uri | unconventional prefoldin RPB5 interactor  32.0 / 1.4 | NA |  | GENE ID: 5668064 AgaP_AGAP010787 | AGAP010787-PA  270 / 2e-70 | GENE ID: 1273231 AgaP_AGAP002737 | AGAP002737-PA  41.2 / 8e-04 | NA |
| 2 | CK1a | f |  | GENE ID: 32221 CkIalpha  489 / 3e-138 | GENE ID: 43673  discs overgrown  401 / 1e-111 | **X** |  | GENE ID: 1271683  AgaP_AGAP000627  564/ 8e-159 | GENE ID: 1278819  AgaP_AGAP003997  290 bits / 1e-78 | **X** |
| 2 | Poly A polymerase | m |  | GENE ID: 49636  hrg | hiiragi  "poly A polymerase"  340 / 0.0 | NA (no second hit) | **2R** |  | GENE ID: 1274089 AgaP_AGAP004244 | 30.0 / 4.3 | GENE ID: 1278426 AgaP_AGAP004798  29.6 / 5.6 | NA (not identified) |
| 2 | L7 | f |  | GENE ID: 34352 RpL7 | Ribosomal protein L7  292 / 2e-79 | GENE ID: 34625 CG5317 | CG5317  117 / 2e-26 | **2L** |  | GENE ID: 1279884 AgaP_AGAP008916 | 312 / 9e-86 | GENE ID: 1269592 AgaP_AGAP007635 75.5 / 2e-14 | **3R** |
| 2 | CG15828 | m,f |  | GENE ID: 34283 CG15828 | CG15828 76.6 / 8e-14 | GENE ID: 35104  kon | kon-tiki  30.8 / 5.0 | **2L** |  | GENE ID: 4577970 AgaP_AGAP008807 | "similar to Drosophila CG15828"  95.5 / 6e-20 | NA (no second hit) | **3R** |
| 2 | Cry1 | f |  | GENE ID: 42305  cry | cryptochrome  418 / 6e-117 | GENE ID: 35322  phr6-4 | (6-4)-photolyase  201 / 1e-51 | **3R** |  | GENE ID: 1281165 AgaP_AGAP001958 | cryptochrome 1  444 / 2e-125 | GENE ID: 1274104 AgaP_AGAP004261 | AGAP004261-PA  227 / 5e-60 | **2R** |
| 2 | Cry2 ^d^ | m, f |  | GENE ID: 35322 phr6-4 | (6-4)-photolyase 494 / 1e-139 | GENE ID: 42305 cry | cryptochrome  341 / 2e-93 | NA |  | GENE ID: 1274104 AgaP_AGAP004261 | cryptochrome 2  910 / 0.0 | GENE ID: 1275500 AgaP_AGAP008651 | AGAP008651-PA  493 / 1e-139 | **2R** |
| 2 | Mck1/  GSK3/  Shaggy | m, f |  | GENE ID: 31248  sgg | shaggy  463 / 1e-130 | GENE ID: 318552  gskt | gasket 395 / 5e-110 | **X** |  | GENE ID: 1274587 AgaP_AGAP004443 479 / 7e-136 | GENE ID: 1275940 AgaP_AGAP004594 142 / 2e-34 | **2R** |
| 2 | rOps2 ^e^ | m |  | GENE ID: 41889  Rh6 | Rhodopsin 6  452 / 2e-127 | GENE ID: 42367  ninaE | neither inactivation nor afterpotential E  425 / 4e-119 | **3R** |  | GENE ID: 4577364 GPROP3 | putative rhodopsin receptor 3  579 / 5e-166 | GENE ID: 4577354 GPROP1 | putative rhodopsin receptor 1  577 / 2e-165 | **2R** |
| 2 | Lsm3 | m, f |  | GENE ID: 42842 LSm3 | CG31184  43.1 / 6e-04 | NA (no second hit) | **3R** |  | NA (no significant similarity) | NA (no significant similarity) | NA |
| 2 | S12 | f |  | GENE ID: 31228  tko | technical knockout  " 40S ribosomal protein S12, mitochondrial”  181 / 2e-46 | GENE ID: 36576 RpS23 | Ribosomal protein S23  45.4 / 2e-05 | **X** |  | GENE ID: 1276710 AgaP_AGAP006038 | 28.9 / 0.65 | GENE ID: 1277920 AgaP_AGAP008032 | 28.5 / 0.85 | NA (not identified) |
| 3 | lark | m, f |  | GENE ID: 38811 lark | CG8597  291 / 1e-78 | GENE ID: 37070 pAbp | polyA-binding protein  63.9 / 4e-10 | **3L** |  | GENE ID: 1270836 AgaP_AGAP011092 | AGAP011092-PA  66.2 / 3e-11 | GENE ID: 3290608 AgaP_AGAP002374 | AGAP002374-PA  64.7 / 7e-11 | NA (not identified) |
| 3 | Globin | m, f |  | GENE ID: 41930 glob1 | globin 1  40.4 / 5e-04 | GENE ID: 31592 CG17717  30.0 / 7.8 | **3R** |  | GENE ID: 1275625 AgaP_AGAP008768 | 29.3 / 4.5 | NA (no second hit) | NA (not identified) |
| 3 | Timeless | m, f |  | GENE ID: 33571 tim | timeless  665 / 0.0 | GENE ID: 41615 timeout | CG7855  37.4 / 1e-04 | **2L** |  | GENE ID: 5668288 AgaP_AGAP008288 |  734 / 0.0 | GENE ID: 1281890 AgaP_AGAP001287 |  32.0 / 0.003 | **3L** |
| 3 | Lipase | m,f |  | GENE ID: 34463 CG31871 | CG31871 51.2 / 4e-06 | GENE ID: 34452 CG18302 | CG18302 47.8 / 4e-05 | NA (not identified) |  | GENE ID: 1281521 AgaP_AGAP001652  32.7 / 0.002 | GENE ID: 1280921 AgaP_AGAP011718  38.5 bits / 0.008 | NA (not identified) |
| 3 | Clock | m, f |  | GENE ID: 38872 Clk | Clock  171 / 9e-52 | GENE ID: 41084 tgo | tango  59.3 / 5e-13 | **3L** |  | GENE ID: 1276380 AgaP_AGAP005711  247 / 1e-79 | GENE ID: 1268258 AgaP_AGAP012873  52.8 / 4e-12 | **2L** |
| 3 | Mbs | m,f |  | GENE ID: 49070 Mbs | Myosin binding subunit  92.4 / 8e-19 | GENE ID: 47718 CG17829 | CG17829  31.6 / 1.7 | **3L** |  | GENE ID: 1277253 AgaP_AGAP006665  97.8 / 7e-21 | GENE ID: 1270178 AgaP_AGAP006901  30.4 / 1.3 | **2L** |
| 3 | Titin | m, f |  | GENE ID: 38384 CG14964 | CG14964  513 / 2e-145 | GENE ID: 43814  bt | bent  171 / 2e-42 | **3L** |  | GENE ID: 1269678 AgaP_AGAP007556  535 / 1e-152 | GENE ID: 1281535 AgaP_AGAP001633  192 / 4e-49 | **2L** |

For *C. marinus* the table gives the mapped genes with the respective linkage group and whether the gene has been mapped to the male (m) or the female (f) informative map. For *D. melanogaster* and *A. gambiae* it gives the gene ID of the best and the second best BLAST hits (blastX) for the respective *C. marinus* sequence, together with the respective bits and e-values, as well as the chromosome arm location of the best hit. If the orthologue could not be identified, no chromosome arm is given.

^a^ Ciliary Opsins (cOpsins) have specific sequence motifs that discriminate them from other insect opsins, which are rOpsins. In *D. melanogaster* there are no cOpsins (Velar*de et a*l. 2005). In *A. gambiae* there are two cOpsins, just as in *C. marinus*; but phylogenetic analysis shows that these were independent duplications, so that there are no pairwise homologies (Figure S1). For the chromosome arm location this does not matter, as both cOpsins of *A. gambiae* are right next to each other. The next best hits in *A. gambiae* – representing rOpsins – are: GENE ID: 1276796 GPROP7 (68.6 / 4e-12) for *C. marinus* cOps1 and GENE ID: 1282006 GPROP6 (98.6 / 5e-21) for *C. marinus* cOps2.

^b^ The *period* gene is not very conserved and thus hard to identify. In *A. gambiae* the best hit and the 4^th^ best hit are annotated in the genome sequence as directly adjacent genes in the same direction. The 4^th^ best hit (Score = 30.4 bits (67), Expect = 1.0, Identities = 12/14, Positives = 13/14, Gaps = 0/14) is annotated as “similar to per”. Both hits are shorter than *per* in other species. Maybe the gene was accidentially fragmented during automated annotation. There is no other locus annotated as *per* in *A. gambiae*. The two hits are on chromosome arm 2R; the 2^nd^, 3^rd^ and 5^th^ best hit are on chromosome arm 2L.

^c^ Timeless 3 does not have an orhologue in *D. melanogaster* or *A. gambiae* (see Table S3 and Figure S3).

^d^ We do not consider the *D. melanogaster* (6-4)-photolyase an orthologue of *cryptochrome2* (*cry2*) (see Figure S2); *cry2* is not present in *D. melanogaster* (Yuan et al. 2007).

^e^ In both *D. melanogaster* and *A. gambiae* there are three *rOpsin* genes that have similarly good blast hits, so that the exact homologue is questionable. However, in both species the three *rOpsins* fall to the same chromosome arm, so that the approximate location can still be inferred.

Literature

Velarde, R. A., C. D. Sauer, K. K. O. Walden, S. E. Fahrbach and H. M. Robertson, 2005 Pteropsin: A vertebrate-like non-visual opsin expressed in the honey bee brain. Insect Biochemistry and Molecular Biology **35:** 1367-1377.

Yuan, Q., D. Metterville, A. D. Briscoe and S. M. Reppert, 2007 Insect cryptochromes: Gene duplication and loss define diverse ways to construct insect circadian clocks. Molecular Biology and Evolution **24:** 948-955.
