# Supplementary material for: Genetic Architecture of Local Adaptation in Lunar and Diurnal Emergence Times of the Marine Midge Clunio marinus (Chironomidae, Diptera)
Source: PLoS One. 2012 Feb 22;7(2):e32092. doi: 10.1371/journal.pone.0032092 (PMC3285202; doi:10.1371/journal.pone.0032092)
Supplement: Table S5 — AFLP primer combinations. (DOC) [file pone.0032092.s008.doc]

**Table S5**

AFLP primer combinations

| **Mse-Primer** | **Eco-Primer** |  |
| --- | --- | --- |
|  | **IRDye 700** | **IRDye 800** |
|  |  |  |
| AA | ATG | ACT |
|  | CGA | AGG |
| AC | AAC | ACA |
|  | ACC | CGC |
|  | ATG | ACT |
|  | TCT | CAT |
| AG | AAC | ACA |
|  | AAG | ACG |
|  | ACC | CGC |
|  | ATG | ACT |
|  | TCT | CAT |
| AT | AAC | ACA |
|  | AAG | ACG |
|  | ACC | CGC |
|  | ATG | ACT |
|  | TAC | GTA |
| CA | ATG | ACT |
|  | TAC | GTA |
| CT | ATG | ACT |
|  | TAC | GTA |
| CTT | AAG | ACG |
| GA | AAC | ACA |
|  | ACC | CGC |
|  | ATG | ACT |
| GC | AAC | ACA |
|  | ATG | ACT |
| GG | AAC | ACA |
|  | AAG | ACG |
|  | ATG | ACT |
|  | CGA | AGG |
| GT | AAC | ACA |
|  | ACC | CGC |
|  | ATG | ACT |
| TA | AAC | ACA |
|  | ACC | CGC |
|  | ATG | ACT |
|  | TCT | CAT |
|  | TAC | GTA |
| TC | AGA | CGC |
| TG | AAC | ACA |
|  | ACC | CGC |
|  | ATG | ACT |
| TT | AAC | ACA |
|  | ATG | ACT |
