# Supplementary material for: Genetic Architecture of Local Adaptation in Lunar and Diurnal Emergence Times of the Marine Midge Clunio marinus (Chironomidae, Diptera)
Source: PLoS One. 2012 Feb 22;7(2):e32092. doi: 10.1371/journal.pone.0032092 (PMC3285202; doi:10.1371/journal.pone.0032092)
Supplement: Table S6 — Clock genes and light receptors. (DOC) [file pone.0032092.s009.doc]

**Table S6**

Clock genes and light receptors

| **Gene** | **Best nr hit**  **(bits / e value)** | **Sequence obtained by** | **Primers** |
| --- | --- | --- | --- |
|  |  |  |  |
| cOps1 ^a^ | Aedes aegypti  GENE ID: 5566410 AaeL_AAEL005373 | opsin  258 / 9e-67 | degenerate PCR | Degenerate PCR:  For 5’ – WSN YTN ACI GTI YTI WSI TAY GAR MGI T – 3’  (Arendt group, EMBL, unpublished)  Rev 5’ – NCK RAA YTG IKT RTT CAT IMM IAC RTA DAT – 3’ (Aren*dt et a*l. 2004)  Nested Rev 5’ – TTC ATN MMI ACR TAD ATD ATI GGR TTR TA – 3’  (Aren*dt et a*l. 2004)  Scoring:  For 5’ – GGGATATTTGGGGATACAATC – 3’  Rev 5’ – ACGTCCGCTTCTCATAGCAT – 3’ |
| cOps2 ^a^ | Aedes aegypti  GENE ID: 5566410 AaeL_AAEL005373 | opsin  343 / 3e-92 | degenerate PCR | Degenerate PCR:  For 5’ – WSN YTN ACI GTI YTI WSI TAY GAR MGI T – 3’  (Arendt group, EMBL, unpublished)  Rev 5’ – NCK RAA YTG IKT RTT CAT IMM IAC RTA DAT – 3’ (Aren*dt et a*l. 2004)  Nested Rev 5’ – TTC ATN MMI ACR TAD ATD ATI GGR TTR TA – 3’  (Aren*dt et a*l. 2004)  Scoring:  For 5’ – AATTTGCTGGAGCTCACGAC – 3’  Rev 5’ – TGAAAAATCCATAGCAAACACAA – 3’ |
| rOps2 | Aedes aegypti  GENE ID: 5568060 AaeL_AAEL006498 | rhodopsin  582 / 4e-164 | degenerate PCR | Degenerate PCR:  For 5’ – GACBGCBTGYGGWACNGA – 3’  Rev 5’ – TAHGGHGTCCAVGCSADGA – 3’  Scoring:  For 5’ – CTTGTCTCATGACTGGAGCAG – 3’  Rev 5’ – TGGAGAGATCTTCGATTTGGTT – 3’ |
| cry1 | Anopheles gambiae  GENE ID: 1281165 AgaP_AGAP001958 | Cryptochrome 1  444 / 7e-123 | degenerate PCR | Degenerate PCR:  For 5’ – CNTAYCARATGTTYYTNCAYACNG – 3’  Rev 5’ – SWNACCCACATCCARTTNCC – 3’  Scoring:  For 5’ – TCAAAATTCATCAGCTTGGTTG – 3’  Rev 5’ – GCCCTTGACGAAACCATAAA – 3’ |
| cry2 ^b^ | Aedes aegypti  GENE ID: 5575633 AaeL_AAEL011967 |  DNA photolyase  918 / 0.0 | degenerate PCR | Degenerate PCR:  For 5’ – GGTTTCGYCATGGDYTRMGMYTVCAYGATAA – 3’  Rev 5’ – CGHACVGAVAGGCADCCRAADCG – 3’  Scoring:  For 5’ – TCCAAATTTTGATAAAATGTCAGGT – 3’  Rev 5’ – TCAACCGGTATCTTCATTACCA – 3’ |
| per | Blattella germanica  gb|AAN02439.2|AF297552_1 circadian clock protein PERIOD  88.6 / 2e-18 | cDNA library | Scoring:  For 5’ – CCGCCTTTTTCTGTCAGTCT – 3’  Rev 5’ – CATCCTCGCTTGTTMCAATCA – 3’ |
| cyc ^c^ | Culex quinquefasciatus ref|XP_001865023.1  circadian protein clock/arnt/bmal/pas  299 / 4e-102 | cDNA library | Scoring:  For 5’ – TTTGATGATGATGACGCAAAA – 3’  Rev 5’ – CGGGGACAGTTAACGTCTTG – 3’ |
| clk | Culex quinquefasciatus ref|XP_001843414.1  circadian locomoter output cycles kaput (clock)  244 / 1e-78 | cDNA library | Scoring:  For 5’ – GGCGAGGGAACTTCATGTTA – 3’  Rev 5’ – CTGCGGGTTCATTTCGTATT – 3’ |
| tim | Aedes aegypti gb|AAY40757.1| TIMELESS  1006 / 0.0 | cDNA library | Scoring:  For 5’ – GCCAAATGACAACCATTTCC – 3’  For 5’ – TTTCCTTTTTGAGGCGATGT – 3’ |
| tim2 | Aedes aegypti  GENE ID: 5572105 AaeL_AAEL009518 | timeout/timeless-2  195 / 3e-48 | cDNA library | Scoring:  For 5’ – TTGATGCCACGTGTGCTAGT – 3’  Rev 5’ – TGTGGATTCGTTGGAACTTG – 3’ |
| tim3 ^d^ | Culex quinquefasciatus  GENE ID: 6031421 CpipJ_CPIJ000660 | timeout/timeless-2  359 / 4e-97 | cDNA library | Scoring:  For 5’ – GTGCCTCCAATTATGGGAAA – 3’  Rev 5’ – TCCATTCAATGCCTTCTTTT – 3’ |
| ck1a | Anopheles gambiae  GENE ID: 1271683 AgaP_AGAP000627  564/ 8e-159 | cDNA library | Scoring:  For 5’ – TCAAAAGAAAGAGTGCGAGTGA – 3’  Rev 5’ – AAAGCGCAAACTTCGACAAT– 3’ |
| Mck1/GSK3/shaggy | Culex quinquefasciatus  GENE ID: 6038164 CpipJ_CPIJ006114 | mck1  489 / 2e-136 | cDNA library | Scoring:  For 5’ – AGTGGCACGCCAATATGC – 3’  Rev 5’ – AAATTGAACAGCGGAGGAAA– 3’ |
| vrille | Anopheles gambiae  GENE ID: 1278160 AgaP_AGAP007801 |  212 / 4e-53 | cDNA library | Scoring:  For 5’ – TGGTGATGGTGACTGAGCTT – 3’  Rev 5’ – CGTTTGGTGAGACTCAAGACC – 3’ |
| lark | Aedes aegypti  GENE ID: 5579044 AaeL_AAEL013982 | RNA binding motif protein 4,lark  304 / 2e-80 | cDNA library | Scoring:  For 5’ – ATAATTATGCCGGGTGCTCA – 3’  Rev 5’ – CTCGTGATGATGGGAAGTCA – 3’ |

^a^ Ciliary opsins have specific parts that allow discrimination from other insect opsins, which are rOpsins.

^b^ The *A. aegypti* “DNA photolyase” likely is a wrongly annotated cryptochrome 2 (see Figure S 2).

^c^ The cyc gene is incompletely annotated as “circadian protein clock/arnt/bmal/pas” in both *A. aegypti* and *C. quinquefasciatus*.

^d^ The tim3 genes in *C. quinquefasciatus* and *A. aegypti* are wrongly annotated as “timeout/tim2”, although another gene (also annotated as “timeout/tim2”) is the true ortholog (see Fig. S3). The fact that in both mosquitoes two different genes received the same name is likely due to automated annotation.

Literature

Arendt, D., K. Tessmar-Raible, H. Snyman, A. W. Dorresteijn and J. Wittbrodt, 2004 Ciliary photoreceptors with a vertebrate-type opsin in an invertebrate brain. Science **306:** 869-871.
